# Design of AFDM Waveform Encryption for LEO Satellite Networks

**DOI:** 10.3390/s26165282

**Published:** 2026-08-20

**Authors:** Muzi Yuan, Honglei Lin, Chunjiang Ma, Pengcheng Ma, Meiting Yu, Xiaomei Tang

**Affiliations:** 1 School of Science, National University of Defense Technology, Changsha 410073, China; ymz@nudt.edu.cn; 2 National Key Laboratory for Positioning, Navigation and Timing Technology, Changsha 410073, China; 3 School of Electronic Science and Technology, National University of Defense Technology, Changsha 410073, China

**Keywords:** affine frequency division multiplexing (AFDM), physical layer encryption, waveform encryption, time-domain obfuscation, low Earth orbit (LEO) satellite, doubly dispersive channel, integrated sensing and communication (ISAC), remote sensing

## Abstract

Low Earth orbit (LEO) satellite downlinks broadcast over wide ground footprints, exposing Earth-observation and remote-sensing sensor data to passive eavesdropping. Affine frequency division multiplexing (AFDM) is a candidate waveform for the doubly dispersive LEO channel and a natural integrated sensing and communication (ISAC) waveform whose delay–Doppler structure supports target parameter estimation; yet existing secure-AFDM schemes act only in the discrete affine Fourier transform (DAFT) parameter domain, leaving the transmitted waveform structurally recognizable. To address this gap, this paper applies time-domain waveform obfuscation to AFDM as physical layer encryption. Using a secret key, the transmitter permutes the inverse-DAFT samples and applies a phase rotation before chirp-periodic-prefix generation; the mask is unitary, so the peak-to-average power ratio is preserved exactly, and the key-holding receiver retains AFDM’s full delay–Doppler sensing capability, while a no-key receiver obtains a dense composite response that destroys target localization (sensing concentration drops from 0 dB to −16.6 dB). Secret pilot phases enable channel estimation at the legitimate receiver while blocking a naive composite-channel attack. Simulations at N=64 and 128 show that a wrong-key eavesdropper achieves uncoded BER within 0.01 of 0.5 across 0–20 dB and that blind Viterbi–Viterbi phase recovery is no more effective under QPSK (BER 0.46–0.48), while the legitimate SNR penalty stays below 0.5 dB. The mask also suppresses AFDM’s internal structure to the AWGN level under AFDM-aware processing. Time-domain obfuscation offers a complementary physical-layer security layer for confidential LEO remote-sensing data downlink and ISAC waveforms.

## 1. Introduction

**Figure 1:**
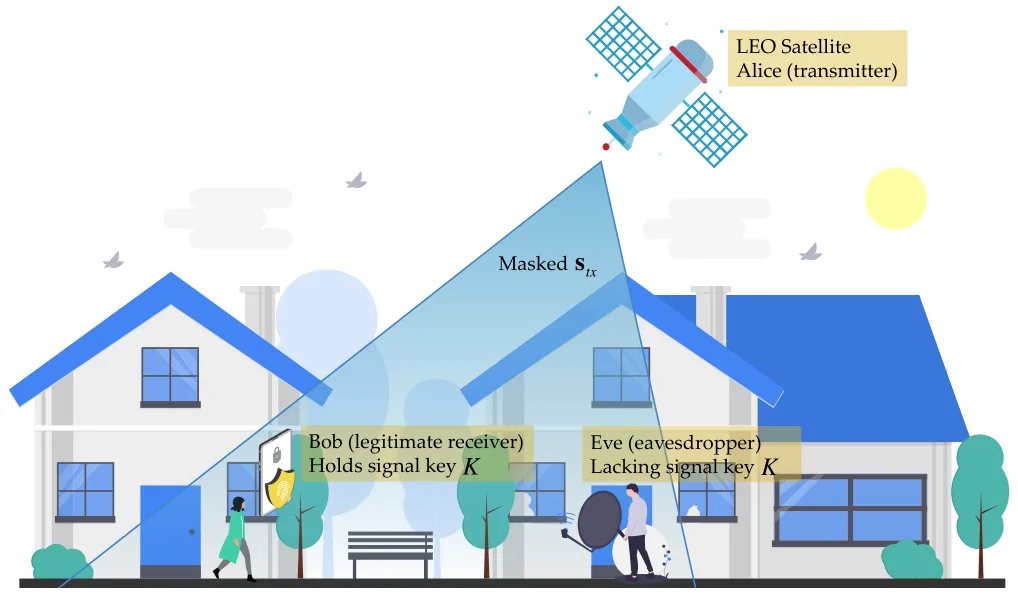
Threat scenario for confidential Earth-observation (EO) data downlink. A LEO satellite broadcasts the AFDM waveform over a wide ground footprint; the proposed scheme masks the inverse-DAFT samples with a key-derived permutation and phase rotation so that the authorized ground station Bob (holding the key *K*) decodes normally, while any passive eavesdropper Eve within the footprint, lacking *K*, fails under the evaluated passive receivers without the key.

Low Earth orbit (LEO) satellite constellations are increasingly deployed as part of next-generation wireless infrastructure, promising global broadband coverage and serving as a non-terrestrial network (NTN) component for 6G networks [1]. Mega-constellations comprising thousands of satellites are being deployed to deliver connectivity to underserved regions, maritime and aeronautical users, and machine-type devices beyond the reach of terrestrial cells, and increasingly to downlink Earth-observation and remote-sensing payload data (imagery, radar products, and telemetry) whose confidentiality can be commercially or operationally sensitive. However, LEO downlinks operate as inherently open broadcast channels: a single satellite illuminates a ground footprint that can span several hundred kilometers, so any receiver within that footprint, whether authorized or not, can capture the transmitted signal. This makes LEO downlinks vulnerable to passive eavesdropping by unauthorized receivers [2], a vulnerability that is qualitatively more severe than in directional terrestrial links. Figure 1 illustrates the resulting threat scenario for confidential Earth-observation data downlink that motivates this work.

Upper-layer cryptography provides mature, standards-based confidentiality for data payloads, and the 5G New Radio NTN inherits the 5G system security architecture and key-management framework [3]; physical layer encryption does not replace it but complements it by addressing a residual waveform-domain exposure that payload encryption leaves open. Payload encryption protects the bits, yet observable features of the physical waveform remain outside payload ciphering and may be exploited for traffic analysis and waveform fingerprinting; for an integrated sensing and communication waveform the exposure is more severe, since a suitably equipped receiver that obtains a waveform reference can run a delay–Doppler sensing receiver on the transmitted signal, so payload confidentiality alone does not protect the sensing function. A key-based transformation applied directly to the waveform samples can obscure the internal sample-level structure of the waveform and the key-derived pilot phases, and degrade target localization for a receiver lacking the key while preserving it for the authorized receiver—a waveform-domain access control effect that payload ciphering does not directly provide. Two physical-layer paradigms pursue this. Physical layer security (PLS) exploits intrinsic properties of the wireless channel—such as reciprocity, fading, and interference—to provide information-theoretic confidentiality guarantees [4,5], but its guarantees typically collapse when the eavesdropper’s channel is as favorable as the legitimate receiver’s. Physical layer encryption (PLE), in contrast, applies key-based transformations directly to the waveform samples [6], providing computational obfuscation that does not depend on a channel advantage; this is the paradigm our scheme belongs to, and its key can be provisioned through the established key-management infrastructure rather than requiring a separate one.

Affine frequency division multiplexing (AFDM) has been studied as a candidate waveform for high-mobility communications in doubly dispersive channels [7,8]. By employing the discrete affine Fourier transform (DAFT), AFDM achieves full diversity in time–frequency doubly selective channels where conventional orthogonal frequency division multiplexing (OFDM) suffers severe inter-carrier interference. These properties make AFDM a candidate waveform for LEO satellite links, which are doubly dispersive: per the 3GPP NTN channel models [9], a 7.5 km/s orbit produces upper-bound line-of-sight Doppler shifts of roughly 50–100 kHz across S-band and 500–750 kHz across Ka-band; we evaluate the scheme under a residual Doppler of up to one subcarrier spacing as a conservative stress-test bound, together with environment-dependent multipath delay spreads. Our link parameters are dimensioned to this regime; Section 3.5 gives an illustrative mapping from NTN quantities to the normalized simulation parameters and scopes a full NTN tapped-delay-line (TDL) realization as future work. This motivates the question of how confidentiality can be built into the AFDM waveform without sacrificing the diversity that motivates its use.

Beyond communications, AFDM is a natural integrated sensing and communication (ISAC) waveform. Its DAFT structure maps each physical scatterer (delay $l_{i}$, Doppler $\alpha_{i}$) to a localized response in the DAFT-domain effective channel, supporting target range and velocity estimation via the delay–Doppler ambiguity function [10,11]. LEO satellites are a primary ISAC platform: the same downlink that delivers Earth-observation and remote-sensing sensor data (imagery, SAR/radar products, telemetry) can simultaneously serve as a sensing waveform for target parameter estimation. Protecting this dual-function link therefore requires that any confidentiality mechanism preserve the sensing capability for the authorized receiver while denying it to an eavesdropper. We show that our unitary time-domain mask achieves exactly this: because permutation and phase rotation are unitary operations, the key-holding receiver recovers the full delay–Doppler sensing response of standard AFDM, while a no-key receiver obtains a dense composite response that destroys target localization (sensing concentration drops from 0 dB to −16.6 dB; Section 4.6).

Existing physical layer protection schemes for AFDM answer this question exclusively in the DAFT or parameter domain, manipulating the chirp parameters $c_{1}$ and $c_{2}$ [10,12,13,14,15]. These schemes share a common limitation: the encrypted waveform remains structurally recognizable as AFDM, and security depends on either channel reciprocity or parameter secrecy. In parallel, Mohammed and Baki [6] proposed Encrypted-OFDM, which applies two-stage time-domain obfuscation (sample scrambling and phase distortion) to OFDM’s inverse fast Fourier transform (IFFT) output, thereby removing the waveform structure rather than hiding a parameter. This time-domain approach has not been applied to chirp-based waveforms such as AFDM, and the adaptation is non-trivial: the DAFT-domain effective channel is not diagonal, the chirp-periodic prefix (CPP) must be preserved after obfuscation, and the receiver architecture must account for the interaction between encryption and the doubly dispersive channel. Section 2 surveys both bodies of work in detail; Table 1 positions our work against them along the axes of operating domain, security basis, security evidence, and overhead. The masking primitive (permutation plus phase rotation after the inverse transform) is inherited from Encrypted-OFDM. This paper adapts it to chirp-based AFDM and characterizes both the operating range and the failure modes. A quantitative head-to-head comparison with chirp-permuted AFDM (CP-AFDM), a representative parameter-domain scheme, is reported in Section 5.14.

This paper investigates time-domain waveform obfuscation for AFDM. The primary methodological novelty is the placement of the encryption mask after the inverse discrete affine Fourier transform (IDAFT) rather than before it. All prior secure-AFDM schemes operate in the DAFT or parameter domain, leaving the transmitted waveform structurally recognizable as AFDM. Our approach moves the mask to the time domain, which hides the waveform structure under the evaluated DAFT-magnitude statistic and forces a completely different receiver design: the effective channel becomes dense, requiring full-band estimation and dense equalization rather than the sparse recovery that parameter-domain schemes rely on. This shift in operating domain is what enables the waveform-level concealment and the secret-pilot defense against composite-channel estimation, neither of which arises naturally in a DAFT-domain scheme. Our main contributions are:

1. We adapt two-stage unitary time-domain obfuscation (sample permutation and phase rotation) to AFDM’s IDAFT output before CPP generation. Because the mask is monomial, the peak-to-average power ratio (PAPR) of the *N* inverse-DAFT samples is preserved exactly (Proposition 1); the CPP-inclusive transmit-block PAPR is measured directly and matches standard AFDM (mean ≈6.72 dB, 99th percentile ≈9.3 dB).
2. We design a full-band secret-pilot channel-estimation scheme and a decrypt-first receiver with a numerically consistent effective channel. The post-decryption effective channel is provably full-rank for every valid key almost surely under the stated path-gain model (Proposition 2), and the secret-pilot estimation-error covariance is invariant under decryption (Lemma 1). With a numerically known effective channel, the legitimate SNR penalty is below 0.5 dB.
3. We empirically characterize the scheme’s obfuscation against the specified class of passive receivers (A1–A4), for which the stated objective is computational obfuscation. A wrong-key eavesdropper on QPSK attains uncoded BER within ∼0.01 of 0.5 across 0–20 dB SNR; blind Viterbi–Viterbi phase recovery is no more effective under QPSK (BER 0.46–0.48); secret pilot phases block the evaluated naive composite-channel estimator; and the AFDM-aware statistic approaches its AWGN-baseline level.
4. We show that the unitary mask preserves AFDM’s delay–Doppler sensing capability for the key-holding receiver while degrading it for the evaluated no-key receiver: sensing concentration at the true target cells is 0 dB for the key holder versus −16.6 dB for the no-key receiver, with 2/4 versus 0/4 targets detectable above a −6 dB threshold.

We state the scope of these contributions explicitly, separating what is demonstrated from what remains open. The demonstrated results above hold under the evaluated passive receivers and the default N=64, QPSK, integer-Doppler regime (with N=128, 16-quadrature amplitude modulation (16QAM), and fractional-Doppler scalability checks). The security boundary is reached by known-plaintext analysis: a multi-frame known-plaintext LS attack recovers the composite channel progressively (BER 0.106 at *M* = *N* = 64, 20 dB), and a single known-plaintext frame carrying known symbols on enough subcarriers fixes the per-subcarrier phases and breaks composite-channel confidentiality, which makes per-frame nonce-derived pilots mandatory; we validate this per-frame-nonce defense against the evaluated cross-frame anchor attack, noting that it prevents cross-frame anchoring but does not remove the same-frame known-plaintext vulnerability. Confidentiality against same-frame known-plaintext and against coded or decision-directed adversaries is not established and is left to upper-layer encryption, consistent with positioning the scheme as a complementary layer. Finally, the evaluation is simulation-based; full 3GPP NTN-TDL channel realization and hardware or over-the-air validation are future work (Section 6). The sample mask R, A are static within a run (not refreshed per frame), so a known data frame compromises subsequent frames regardless of pilot nonces; full per-frame defense requires refreshing the data mask as well.

Section 2 reviews the physical layer encryption and security, AFDM, and time-domain encryption and the LEO/NTN context. Section 3 presents the materials and methods: AFDM fundamentals, the doubly dispersive channel and effective-channel construction, the system and threat models, the two-stage time-domain obfuscation design, the secret-pilot training scheme, and the decrypt-first receiver architecture. Section 4 analyzes the security properties, formalizes the obfuscation objective and adversary classes, and states the limitations. Section 5 presents simulation results across fourteen experiment blocks. Section 6 discusses deployment considerations, composability with parameter-domain methods, standardization, and limitations and future work. Section 7 concludes.

## 2. Related Work and Background

We review three relevant areas, namely physical layer encryption (PLE) and physical layer security (PLS), the AFDM waveform and its recent secure variants, and time-domain waveform encryption. We also summarize the non-terrestrial network (NTN) context that motivates the LEO downlink threat model. Table 1 (Section 1) gives a compact comparison; here we expand on the distinctions and, where relevant, the historical lineage of each idea.

### 2.1. Physical Layer Protection: From Information-Theoretic Secrecy to Key-Based Encryption

Physical layer protection encompasses two complementary paradigms distinguished by their security basis: physical layer security (PLS) and physical layer encryption (PLE).

Physical layer security [4,5] exploits intrinsic channel characteristics—tracing to Shannon’s perfect secrecy and Wyner’s wiretap channel—to establish a positive secrecy capacity when the legitimate channel is stochastically stronger than the eavesdropper’s [16,17]. It is fundamentally channel-advantage dependent: the guarantees collapse when Eve’s channel is as good as or better than Bob’s. Recent PLS work has extended this paradigm to integrated sensing and communication. Illi et al. [18] jointly optimize secrecy and sensing in reconfigurable intelligent surface (RIS)-assisted full-duplex ISAC networks, maximizing sensing performance under secrecy and power constraints. Their approach targets malicious sensing targets rather than passive eavesdroppers and relies on information-theoretic secrecy rather than computational obfuscation.

Physical layer encryption departs from this model by applying key-based transformations directly to the waveform samples [6,19]. Rather than relying on a channel advantage, PLE uses a secret key shared between Alice and Bob (derived from reciprocal channel measurements [20] or pre-distributed out-of-band) to scramble the transmitted waveform so that an adversary without the key cannot recover the data. Security is computational rather than information-theoretic. It is tied to the adversary’s inability to invert the key-dependent transformation, and does not degrade when the eavesdropper’s channel improves. This is the paradigm our scheme belongs to.

For the purposes of this paper, it is useful to organize physical layer protection techniques along an additional axis, the operating domain: protection may be introduced in the information/bit domain (secrecy coding), the modulation/constellation domain (symbol scrambling, directional modulation), the frequency/parameter domain (subcarrier permutation, chirp-parameter modulation), or the time-sample domain (post-transform sample masking). Our work is a PLE, time-sample-domain scheme; this places it in a region of the design space that, for chirp waveforms, has been essentially unexplored.

A key-based sample-masking scheme of the kind we study provides computational obfuscation against a specified receiver class, not information-theoretic secrecy. We make no secrecy capacity claim, and our security evidence is uncoded bit error rate (BER) under explicitly stated adversaries, which is a necessary but far from sufficient indicator (Section 4.1).

### 2.2. AFDM and Chirp-Based Waveforms

Doubly dispersive channels (those exhibiting both time selectivity from Doppler and frequency selectivity from delay spread) are the defining challenge of high-mobility links such as high-speed rail, vehicular, aeronautical, and LEO satellite communications. Orthogonal frequency division multiplexing (OFDM), the workhorse of 4G/5G, loses its orthogonality under Doppler and suffers inter-carrier interference, motivating a family of next-generation waveforms. Orthogonal time–frequency space (OTFS) modulation multiplexes symbols in the delay–Doppler domain and achieves full diversity in such channels [21,22]. Frequency-modulated OFDM [23] offers another chirp-based alternative. Affine frequency division multiplexing (AFDM), introduced by Bemani et al. [7,8], achieves comparable full-diversity performance using a single chirp-multiplexing transform (the discrete affine Fourier transform, DAFT) with a lower-dimensional implementation [24]. The two chirp parameters $c_{1}$ and $c_{2}$ are chosen so that the delay–Doppler paths map to distinct, non-overlapping locations in the DAFT domain, yielding a sparse effective channel and enabling low-complexity detection. Because AFDM’s chirp structure matches the geometry of doubly dispersive channels, it is an attractive downlink waveform for LEO satellites, where large Doppler shifts and multipath delays coexist. Our scheme is deliberately waveform-preserving: it wraps AFDM without altering the chirp parameters, so that AFDM’s BER performance and receiver structure are retained under the tested uncoded settings for the legitimate receiver.

### 2.3. Secure AFDM in the Parameter Domain

Because the DAFT is parameterized by $c_{1}$ and $c_{2}$, a natural route to secure AFDM is to treat these parameters, or a generalization of them, as a secret. This line of work has developed along a discernible trajectory over the past two years, moving from channel-dependent parameter selection toward increasingly general parameter-domain transformations.

The trajectory begins with Tek and Başar [12], who vary $c_{2}$ as a function of the legitimate channel state, so only a receiver whose channel matches can demodulate—a channel-advantage scheme whose protection erodes if Eve’s channel correlates with Bob’s. Wang et al. [13] (SE-AFDM) instead drive $c_{2}$ with a secret low-probability-of-detection spreading sequence, targeting confidentiality and covertness via parameter secrecy. Rou and de Abreu [14] (CP-AFDM, the closest prior work) permute the chirp subcarriers themselves—the same permutation primitive we use, but in the DAFT rather than the time domain, so the transmitted signal retains recognizable per-sample chirp structure. Xu et al. [25] analyze parameter-design vulnerabilities. Cao et al. [10] extend the parameter-domain approach to multi-layer encryption in multiple-input multiple-output (MIMO)-AFDM with index modulation, while Zhang et al. [15] design secure AFDM waveforms for satellite-air-integrated links, and Zhang and Dai [11] propose a security-aware codebook that simultaneously addresses eavesdropping and PAPR. These schemes share a defining trait: the waveform remains a chirp-multiplexed AFDM signal whose chirp-rate and cyclostationary signatures stay recognizable, because protection never leaves the DAFT domain. Our approach is orthogonal: we leave $c_{1},c_{2}$ public and mask the time-domain samples after the inverse DAFT—and is composable with the parameter-domain family (Section 6). We report a head-to-head simulation comparison with CP-AFDM in Section 5.14; Table 1 remains qualitative for the other baselines, and Section 6.4 analyzes the parameter-domain/time-domain tradeoff.

### 2.4. Physical Layer Encryption via Time-Domain Waveform Masking

The paradigm of physical layer encryption (PLE)—applying key-based transformations directly to waveform samples rather than exploiting channel properties—has a short but growing history in the OFDM literature, appearing under labels such as encrypted-OFDM, chaotic/scrambled OFDM, and constellation/phase obfuscation. The most directly relevant precedent is Encrypted-OFDM of Mohammed and Baki [6], which applies a two-stage time-domain transformation (sample scrambling via permutation followed by phase distortion) to the IFFT output of OFDM, reporting near-0.5 uncoded BER for a wrong-key eavesdropper. The primitive we adopt (permutation plus per-sample phase rotation after the inverse transform) is inherited directly from that line of work, and we credit it as such.

What does not transfer from OFDM to AFDM is the receiver theory, and this is where the adaptation is non-trivial. In OFDM the per-subcarrier channel is diagonal, so a post-IFFT time-domain mask leaves an easily invertible structure and channel estimation is per-subcarrier least squares. In AFDM the effective DAFT-domain channel is non-diagonal: a sparse shifted-diagonal matrix with at most *P* nonzeros per row under integer Doppler, and fully spread under fractional Doppler. Three specific complications follow, none of which arise in Encrypted-OFDM: (i) the chirp-periodic prefix (CPP), rather than a plain cyclic prefix, must be generated after masking so that the chirp-periodic property survives; (ii) after masking, the one-nonzero-per-row sparsity of the AFDM channel is destroyed, so the receiver must estimate and invert a dense effective channel; and (iii) naive per-subcarrier pilot estimation fails, requiring the full-band secret-pilot scheme we develop in Section 3.7. We contribute this adaptation and a candid security characterization, including the discovery (Section 4.5) that a single known-plaintext frame breaks composite-channel confidentiality unless per-frame nonces are used, a failure mode not identified in the OFDM precedent.

### 2.5. LEO/NTN Security Context

Non-terrestrial networks are central to 6G research and to 3GPP Release-17 and later work on New Radio (NR) support for NTN [1,9]. LEO constellations, orbiting at 300–1500 km, deliver low-latency broadband but broadcast over a footprint that can span hundreds of kilometers, so any receiver within that footprint, whether authorized or not, can capture the downlink. This wide-footprint broadcast property makes eavesdropping markedly easier than in a directional terrestrial link and is the core motivation for PLE-based confidentiality in this setting [2]. Two further NTN characteristics shape the design. First, the doubly dispersive channel (large Doppler from ∼7.5 km/s orbital velocity, multipath from ground scattering) is exactly the regime AFDM targets, so a security layer must not disturb AFDM’s diversity. Second, the link already carries periodic signaling for Doppler pre-compensation and beam/handover management. Our scheme requires per-frame pilot-phase freshness (Section 4.4), a per-frame synchronization burden comparable to this existing signaling; secure nonce synchronization can be built on the same frame counter but is not provided by Doppler or handover signaling alone. These observations frame both the opportunity and the constraints for the scheme developed next.

## 3. Materials and Methods

### 3.1. AFDM Waveform Fundamentals

Table 2 collects the principal symbols used throughout the paper; they are introduced formally where they first appear.

AFDM is a chirp-based multicarrier waveform designed for reliable communication in doubly dispersive (time–frequency selective) channels [7,8]. Unlike OFDM, which modulates symbols onto orthogonal sinusoids via the discrete Fourier transform (DFT), AFDM employs the discrete affine Fourier transform (DAFT), which uses chirp carriers parameterized by two real-valued parameters $c_{1}$ and $c_{2}$.

Let $x={\left[x\left[0\right],x\left[1\right],\ldots ,x\left[N-1\right]\right]}^{⊤}\in C^{N\times 1}$ denote the vector of *N* complex-valued data symbols in the DAFT domain. The time-domain AFDM signal $s={\left[s\left[0\right],\ldots ,s\left[N-1\right]\right]}^{⊤}$ is obtained via the inverse DAFT (IDAFT):

(1) $$s\left[n\right]=\frac{1}{\sqrt{N}}\sum_{m=0}^{N-1}x\left[m\right]\;\exp\;\left(j2\pi \left(c_{1}n^{2}+\frac{nm}{N}+c_{2}m^{2}\right)\right),$$

for n=0,1,…,N−1. In compact matrix form:

(2) $$s=\Lambda_{c_{1}}^{H}F^{H}\Lambda_{c_{2}}^{H}x,$$

where F is the normalized *N*-point DFT matrix with entries $F_{k,n}=\frac{1}{\sqrt{N}}e^{-j2\pi kn/N}$, and the diagonal chirp matrices are:

(3) $$\begin{array}{cc} \Lambda_{c_{1}} & =\mathrm{diag}\;\left(e^{-j2\pi c_{1}\cdot 0^{2}},\ldots ,e^{-j2\pi c_{1}\cdot {\left(N-1\right)}^{2}}\right), \end{array}$$

(4) $$\begin{array}{cc} \Lambda_{c_{2}} & =\mathrm{diag}\;\left(e^{-j2\pi c_{2}\cdot 0^{2}},\ldots ,e^{-j2\pi c_{2}\cdot {\left(N-1\right)}^{2}}\right). \end{array}$$

The chirp parameters are chosen to guarantee full diversity in doubly dispersive channels. Following [7]:

(5) $$c_{1}=\frac{2\alpha_{\max}+1}{2N},\;c_{2}=\frac{\sqrt{2}}{N},$$

where $\alpha_{\max}\in Z^{+}$ is the maximum integer Doppler shift normalized by the subcarrier spacing $\Delta f=1/(NT_{s})$, and $T_{s}$ is the sampling period. The irrational choice $c_{2}=\sqrt{2}/N$ ensures that the DAFT basis functions are linearly independent under any doubly dispersive channel with integer Doppler shifts, guaranteeing full diversity [8].

The DAFT operator $D\left(\cdot \right)≜\Lambda_{c_{2}}F\Lambda_{c_{1}}$ is unitary, being a product of the unitary DFT matrix F and two unit-modulus diagonal matrices; hence $D^{-1}=D^{-1}=D^{H}$ preserves energy and whitens noise. Setting $c_{1}=c_{2}=0$ reduces D to the ordinary DFT and AFDM to OFDM, so AFDM is a strict generalization of OFDM in which the two chirp parameters introduce a quadratic phase pre- and post-rotation. The role of $c_{1}$ is to shear the delay–Doppler plane so that paths with distinct $\left(l_{i},\alpha_{i}\right)$ map to distinct DAFT-domain locations (9), while the irrational $c_{2}$ breaks residual degeneracies to secure full diversity. Relative to OTFS [21,22], which spreads each symbol over the entire delay–Doppler plane using a two-dimensional transform, AFDM attains comparable diversity with a single one-dimensional chirp transform and a lower-complexity implementation [24], which is one reason we adopt it as the base waveform.

**Remark** **1.** *Our obfuscation acts on the time-domain samples $s=D^{-1}\left(x\right)$, i.e., after the chirp transform. This location is deliberate. Acting before the IDAFT (in the DAFT domain) would amount to a parameter/symbol-domain scheme of the kind already studied (Section 2) and would leave the transmitted samples chirp-structured. Acting after the IDAFT masks the per-sample chirp signature while, because the transform is unitary and known to Bob, leaving the legitimate receiver a clean linear inverse. The cost is that the post-encryption effective channel is no longer sparse, which drives the receiver and estimation design of Section 3.7.*

### 3.2. Chirp-Periodic Prefix

To combat inter-block interference, AFDM employs a chirp-periodic prefix (CPP) of length $L_{\mathrm{cp}}\geq l_{\max}$, where $l_{\max}$ is the maximum channel delay spread in samples. Unlike the standard cyclic prefix in OFDM, the CPP applies a phase correction to ensure the prefix samples satisfy the chirp-periodic property:

(6) $$s_{\mathrm{tx}}\left[n\right]=s\left[N+n\right]\;e^{-j2\pi c_{1}\left(N^{2}+2Nn\right)},\;n=-L_{\mathrm{cp}},\ldots ,-1.$$

The CPP-prepended signal is $s_{\mathrm{tx}}={\left[s_{\mathrm{tx}}\left[-L_{\mathrm{cp}}\right],\ldots ,s_{\mathrm{tx}}\left[-1\right],s\left[0\right],\ldots ,s\left[N-1\right]\right]}^{⊤}\in C^{(N+L_{\mathrm{cp}})\times 1}$. After propagation through the channel and CPP removal at the receiver (discarding the first $L_{\mathrm{cp}}$ samples), the effective time-domain input–output relationship becomes a circulant-like convolution, which diagonalizes in the DAFT domain. The CPP is constructed from the tail of the data block via the chirp-periodic correction (6), regardless of whether the tail samples carry encrypted or unencrypted magnitudes. The chirp-periodic property is a structural property of the prefix construction, not of the data values it copies. Encryption changes which magnitudes occupy the last $L_{\mathrm{cp}}$ samples, but the phase correction $e^{-j2\pi c_{1}\left(N^{2}+2Nn\right)}$ in (6) is applied identically. Multipath with maximum delay $l_{\max}\leq L_{\mathrm{cp}}$ is absorbed by the prefix, yielding the circular convolution structure needed for DAFT-domain diagonalization. The randomized magnitudes in the prefix do not break this structure because the chirp-periodic correction operates on the sample values, not on their statistics.

### 3.3. Doubly Dispersive Channel Model

We consider a baseband equivalent doubly dispersive multipath channel with *P* propagation paths [7]. For path i∈{1,…,P}, let $h_{i}\in C$, $\tau_{i}$, and $\nu_{i}$ denote the complex gain, delay, and Doppler shift, respectively. In discrete time, the delays become integer multiples of $T_{s}$: $\tau_{i}=l_{i}T_{s}$ with $l_{i}\in \left\{0,1,\ldots ,l_{\max}\right\}$, and the Doppler shifts become normalized: $\alpha_{i}=\nu_{i}/\Delta f\in \left[-\alpha_{\max},\alpha_{\max}\right]$.

After CPP removal, the received time-domain samples are:

(7) $$r\left[n\right]=\sum_{i=1}^{P}h_{i}\;e^{j2\pi \alpha_{i}n/N}\;s\left[\left(n-l_{i}\right)\;\mathrm{mod}\;N\right]+w\left[n\right],$$

for n=0,1,…,N−1, where $w\left[n\right]∼\mathrm{CN}\left(0,\sigma^{2}\right)$ is independent circularly symmetric complex Gaussian noise with variance $\sigma^{2}=1/{\mathrm{SNR}}_{\mathrm{lin}}$, and ${\mathrm{SNR}}_{\mathrm{lin}}=10^{{\mathrm{SNR}}_{\mathrm{dB}}/10}$ is the linear SNR normalized to unit average signal power.

When $\alpha_{i}\in Z$ for all *i*, the DAFT of the received signal yields:

(8) $$y=D\left(r\right)=\Lambda_{c_{2}}F\Lambda_{c_{1}}r=H_{\mathrm{eff}}\;x+\tilde{n},$$

where $y\in C^{N\times 1}$ is the DAFT-domain received vector, $\tilde{n}=D\left(w\right)$ is the DAFT-domain noise (still white with variance $\sigma^{2}$ since the DAFT is unitary), and $H_{\mathrm{eff}}\in C^{N\times N}$ is the effective channel matrix. For integer Doppler with paths at distinct locations ${\mathrm{loc}}_{i}$, each path contributes one nonzero per row to $H_{\mathrm{eff}}$, yielding a sparse shifted-diagonal structure with at most *P* nonzeros per row [7]: each DAFT-domain subcarrier receives a contribution from exactly one transmitted subcarrier, with the shift determined by the path-dependent location:

(9) $${\mathrm{loc}}_{i}=\left(2Nc_{1}l_{i}-\alpha_{i}\right)\;\mathrm{mod}\;N.$$

The nonzero entry in row *p* and column $q=(p+{\mathrm{loc}}_{i})\;\mathrm{mod}\;N$ is:

(10) $${\left[H_{\mathrm{eff}}\right]}_{p,q}=\sum_{i:{\mathrm{loc}}_{i}=\left(q-p\right)\;\mathrm{mod}\;N}h_{i}\;\exp\;\left(j\frac{2\pi }{N}\left(Nc_{1}l_{i}^{2}-ql_{i}+Nc_{2}\left(q^{2}-p^{2}\right)\right)\right).$$

The one-nonzero-per-row structure follows from conjugating the cyclic-shift operator by the chirp diagonal $\Lambda_{c_{1}}$, which produces a linear phase ramp that the DFT diagonalizes into the frequency shift ${\mathrm{loc}}_{i}$ of (9). Critically, this argument assumes the input propagates through $D^{-1}$ without intermediate transformations; inserting the encryption between $D^{-1}$ and the channel destroys the conjugation structure, making the post-encryption channel typically effectively dense and requiring the full-band estimation of Section 3.7.

When the normalized Doppler shifts contain fractional components $\alpha_{i}=\alpha_{i}^{\mathrm{int}}+\nu_{i}$ with $\nu_{i}\in \left[-\xi ,\xi \right]$ and ξ∈[0,0.5], the one-nonzero-per-row sparsity is lost. Each path contributes energy to multiple adjacent DAFT subcarriers, with the spreading controlled by ξ. The guard symbol requirement increases to $\alpha_{\max}+\left\lceil \xi \right\rceil$ per side to suppress inter-DAFT-block interference.

**Numerical $H_{\mathrm{eff}}$ construction.** For our simulations, we construct $H_{\mathrm{eff}}$ numerically by probing each DAFT subcarrier individually. For subcarrier *p*, we transmit a one-hot DAFT vector $x=e_{p}$, compute the IDAFT to obtain the time-domain signal, add CPP, apply the exact time-domain channel (7) without noise, remove CPP, and apply the DAFT. The resulting vector is the *p*-th column of $H_{\mathrm{eff}}$; the procedure is summarized in Algorithm 1. This numerical construction guarantees perfect consistency between the channel simulator and the linear minimum mean-square error (LMMSE) equalizer, avoiding the analytical phase mismatch issues we identified during our implementation [7]. The computational cost is $O(N^{2}\cdot P)$ per channel realization, which is acceptable for offline simulation and can be accelerated via pre-computation of the DAFT basis.
**Algorithm 1** Numerical effective channel matrix construction.
**Input:** Channel parameters ${\left\{h_{i},l_{i},\alpha_{i}\right\}}_{i=1}^{P}$, AFDM modem
**Output:** $H_{\mathrm{eff}}\in C^{N\times N}$1:**for** 
p=1 **to** *N* **do**2:       $x\leftarrow e_{p}$▷ One-hot DAFT vector3:       $s\leftarrow D^{-1}\left(x\right)$▷ Time-domain signal4:       $s_{\mathrm{tx}}\leftarrow \mathrm{add}\text{\_}\mathrm{cpp}\left(s\right)$▷ Add CPP5:       $r\leftarrow 0\in C^{N+L_{\mathrm{cp}}}$6:       **for** i=1 **to** *P* **do**7:             $r\leftarrow r+h_{i}\cdot e^{j2\pi \alpha_{i}n/N}\cdot \mathrm{shift}\left(s_{\mathrm{tx}},l_{i}\right)$8:       **end for**9:       $r\leftarrow r[L_{\mathrm{cp}}:N+L_{\mathrm{cp}}-1]$▷ Remove CPP10:       $H_{\mathrm{eff}}\left[:,p\right]\leftarrow D\left(r\right)$11:**end for**

### 3.4. Channel Estimation and Equalization

With perfect channel state information (CSI) at the receiver (the “genie-aided” case), Bob employs LMMSE equalization in the DAFT domain:

(11) $$\hat{x}={\left(H_{\mathrm{eff}}^{H}H_{\mathrm{eff}}+\sigma^{2}\lambda I_{N}\right)}^{-1}H_{\mathrm{eff}}^{H}y,$$

where λ=1 (standard LMMSE, with the regularization matching the noise variance $\sigma^{2}$). The equalized DAFT-domain symbols are then restricted to the active data subcarriers $I_{\mathrm{data}}=\left\{\alpha_{\max}+1,\ldots ,N-\alpha_{\max}\right\}$ and passed to the QAM demodulator.

For practical channel estimation, AFDM typically employs pilot symbols placed on a subset of DAFT subcarriers [7]. Because $H_{\mathrm{eff}}$ is not diagonal (each received subcarrier maps to a different transmitted subcarrier), per-subcarrier least-squares (LS) estimation fails. The conventional solution is to estimate the path parameters ${\left\{h_{i},l_{i},\alpha_{i}\right\}}_{i=1}^{P}$ via sparse recovery and reconstruct $H_{\mathrm{eff}}$ from (10). This approach requires knowledge of the pilot structure and is sensitive to fractional Doppler. In Section 3.7, we design a practical full-band pilot-based channel estimation scheme that integrates with the encrypted waveform.

### 3.5. LEO Satellite Channel Characteristics

The LEO relevance of the scheme rests on the doubly dispersive nature of the satellite downlink, which we characterize using the quantities defined in the 3GPP non-terrestrial-network (NTN) channel models [9]. Table 3 then gives an illustrative dimensional mapping from these NTN-motivated quantities to the normalized AFDM simulation parameters. This is a dimensional interpretation rather than a standards-compliant NTN link realization; a full NTN-TDL channel study is left to future hardware-validated work (Section 6).

LEO satellite downlinks operate at altitudes of 300–1500 km with orbital velocities of approximately 7.5 km/s. The resulting raw Doppler shift is approximately 50–100 kHz across S-band (2–4 GHz) and 500–750 kHz across Ka-band (20–30 GHz) as upper-bound line-of-sight magnitudes. In practice, ephemeris-aided Doppler pre-compensation at the satellite or ground terminal removes the bulk of this shift. We adopt $\alpha_{\max}=1$ as a conservative stress-test bound: we evaluate the scheme under a residual Doppler of up to one subcarrier spacing, chosen so that the security and sensing results are not artifacts of an optimistic Doppler assumption.

The normalized parameters $\alpha_{\max}=1$ and $l_{\max}=6$ are the doubly dispersive scales our simulations exercise—a residual Doppler of up to one subcarrier spacing and a multipath excess delay of up to six samples. Interpreted at a representative instantaneous bandwidth B=NΔf, these correspond to a residual Doppler of Δf and a maximum excess delay of 6/B. For illustration, at a Ka-band, ≈15 MHz design point with the NR numerology Δf=240 kHz (used here for dimensioning only, not as an NTN data allocation), this is a residual Doppler of 240 kHz and a maximum modeled excess delay of about 390 ns; the RMS delay spread of the profile below at this scale is about 64 ns. The 5G NR subcarrier spacings span 15–240 kHz, and TR 38.811 [9] (surveyed in [1]) provides the NTN tapped-delay-line modeling framework from which these scales are motivated.

Our simulations adopt a representative 6-tap exponentially decaying power delay profile with path losses [0,−3.6,−7.2,−10.8,−18.0,−25.2] dB and delays [0,1,2,3,5,6] samples, matching the parameters used in [7] for $l_{\max}=6$. The Doppler shifts are uniformly drawn from $\left[-\alpha_{\max},\alpha_{\max}\right]$ for integer Doppler experiments, and additionally perturbed by U(−ξ,ξ) for fractional Doppler experiments. This representative profile is not a 3GPP NTN-TDL realization; it captures the essential doubly dispersive characteristics—a bounded residual Doppler and a multipath excess delay within the CPP guard—on which the security and sensing mechanisms of this paper rely, and it is the channel on which algorithmic feasibility is established.

### 3.6. Threat Model

We adopt a conservative wiretap channel model with three parties, namely a legitimate transmitter (Alice), a legitimate receiver (Bob), and a passive eavesdropper (Eve). Alice and Bob share a pre-established secret key K∈K, obtained through an authenticated key agreement protocol whose design is outside the scope of this paper.

Alice (the satellite) modulates data symbols $x\in C^{N\times 1}$ using the AFDM waveform, applies time-domain obfuscation ${\mathrm{Enc}}_{K}\left(\cdot \right)$ (described in Section 3.7), adds CPP, and transmits the resulting signal $s_{\mathrm{tx}}$ on the downlink.

Bob receives $r_{B}=H_{B}\left(s_{\mathrm{tx}}\right)+w_{B}$, where $H_{B}$ is the legitimate channel operator as defined in (7). Bob knows *K* and can therefore decrypt and demodulate the signal.

We grant Eve every advantage short of the secret key:

1. System knowledge: Eve knows all AFDM parameters (*N*, $c_{1}$, $c_{2}$, $\alpha_{\max}$, $l_{\max}$, $L_{\mathrm{cp}}$, the modulation format, and the frame structure). She also knows the encryption algorithm (but not the key *K*).
2. Channel knowledge: Eve has perfect CSI for her own channel $H_{E}$ (genie-aided $H_{\mathrm{eff}}^{\left(E\right)}$), and her receive SNR may be higher than Bob’s (e.g., if she is closer to the satellite).
3. Pilot knowledge: Eve knows the pilot structure and can observe all pilot transmissions. However, she does not know the secret pilot phases $\left\{\theta_{p}\right\}$ derived from *K*.
4. Multi-frame analysis: Eve can passively collect multiple frames and perform offline statistical and algebraic analysis across them.
5. Known-plaintext access: Eve may know a subset of the transmitted data symbols, e.g., from standardized protocol headers, synchronization words, or predictable traffic patterns.
6. Computational resources: Eve has polynomially bounded computational resources (she cannot brute-force the full key space).

This threat model is deliberately conservative, stress-testing the scheme within the enumerated passive model. Any security demonstrated under these assumptions defines a conservative evaluation of the obfuscation layer, as a real-world Eve is unlikely to simultaneously enjoy perfect CSI, higher SNR than Bob, known plaintext, and multi-frame collection capability.

### 3.7. Proposed Encrypted-AFDM: Two-Stage Time-Domain Encryption

**Figure 2:**
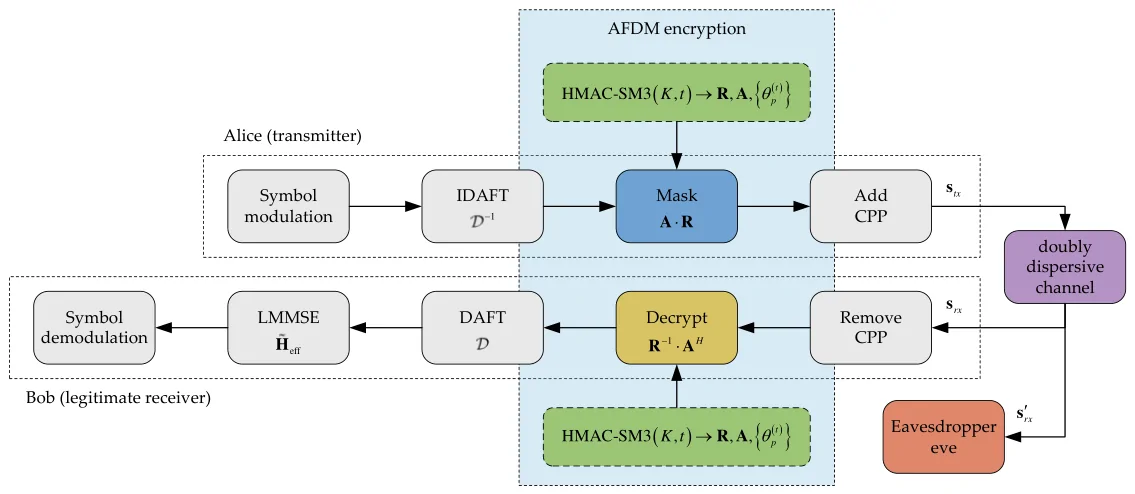
Encrypted-AFDM signal chain. The two-stage time-domain mask AR is applied to the IDAFT output before CPP generation; Bob inverts it with $R^{-1}A^{H}$ before DAFT demodulation and LMMSE equalization. The permutation, phase mask, and per-frame secret pilot phases are all derived from the shared key *K* (and, for a deployed system, a frame nonce *t*) via HMAC-SM3 (RFC 2104); in our current simulations, R and A are static, key-derived but not frame-dependent. A passive eavesdropper (omitted from the figure for clarity) observes $s_{\mathrm{tx}}$ through her own channel but lacks *K*.

The core idea of Encrypted-AFDM is to encrypt the AFDM waveform at the sample level in the time domain, after IDAFT modulation but before CPP generation. This differs from prior secure-AFDM schemes that modify DAFT-domain parameters. Figure 2 shows the complete signal chain. The encryption consists of two unitary stages, both derived from a shared secret key *K*:

**Definition** **1.** *Given a secret key K, the encryption mapping ${\mathrm{Enc}}_{K}:C^{N}\to C^{N}$ is:*

(12) $$s_{\mathrm{enc}}={\mathrm{Enc}}_{K}\left(s\right)=A\cdot R\cdot s,$$

*where:*

- *$R\in {\left\{0,1\right\}}^{N\times N}$ is a permutation matrix (sample scrambling) derived from K via a pseudo-random permutation of {1,…,N}.*
- *$A=\mathrm{diag}(e^{j\phi_{1}},e^{j\phi_{2}},\ldots ,e^{j\phi_{N}})$ is a diagonal phase rotation matrix with $\phi_{n}\in \left[0,2\pi \right)$ drawn pseudo-randomly from K.*

Both R and A are unitary: $R^{-1}=R^{⊤}$ and $A^{-1}=A^{H}$. Decryption consists of the inverse operation:

(13) $${\mathrm{Dec}}_{K}\left(s_{\mathrm{enc}}\right)=R^{-1}\cdot A^{H}\cdot s_{\mathrm{enc}}=s.$$

The encryption is applied to the IDAFT output $s=D^{-1}\left(x\right)$ before CPP addition. The complete transmitter chain is:

(14) $$x\overset{D^{-1}}{\to }s\overset{{\mathrm{Enc}}_{K}}{\to }s_{\mathrm{enc}}\overset{\mathrm{CPP}}{\to }s_{\mathrm{tx}},$$

where $s_{\mathrm{tx}}\in C^{(N+L_{\mathrm{cp}})\times 1}$ is the transmitted signal with CPP.

Encrypting before CPP generation is critical: the CPP Formula (6) operates on the encrypted samples, ensuring the chirp-periodic property is preserved in the transmitted signal. If encryption were applied after CPP, the chirp-periodic relationship would be destroyed, breaking AFDM’s full diversity.

The permutation R reorders samples and the diagonal A applies unit-modulus factors, so the multiset of sample magnitudes $\left\{\right|s_{\mathrm{enc}}\left[n\right]{\left|\right\}}_{n=0}^{N-1}$ equals ${\left\{|s\left[n\right]|\right\}}_{n=0}^{N-1}$. Consequently, the PAPR of the *N* inverse-DAFT samples is preserved exactly.

**Proposition** **1.** *For any $s\in C^{N}$ and any encryption key K,*

(15) $$\mathrm{PAPR}\left(s_{\mathrm{enc}}\right)=\frac{\max_{n}{\left|s_{\mathrm{enc}}\left[n\right]\right|}^{2}}{\frac{1}{N}\sum_{n}{\left|s_{\mathrm{enc}}\left[n\right]\right|}^{2}}=\frac{\max_{n}{\left|s\left[n\right]\right|}^{2}}{\frac{1}{N}\sum_{n}{\left|s\left[n\right]\right|}^{2}}=\mathrm{PAPR}\left(s\right).$$

**Proof.**  $|s_{\mathrm{enc}}\left[n\right]|=|e^{j\phi_{n}}s\left[\pi \left(n\right)\right]|=|s\left[\pi \left(n\right)\right]|$ for the key-derived permutation π, so both the maximum and the average of $|s_{\mathrm{enc}}{\left[n\right]|}^{2}$ are invariant under R and A.    □

**Remark** **2.** *Proposition 1 concerns the N post-IDAFT samples. The transmitted block additionally contains the $L_{\mathrm{cp}}$ CPP samples *(6)*, which repeat a key-dependent subset of the encrypted tail with a unit-modulus chirp correction; the permutation therefore changes which magnitudes are copied into the prefix, so the transmit-block PAPR is not guaranteed to be identical sample-for-sample. Our PAPR experiment (Section 5) measures the PAPR of the N post-IDAFT samples and finds it identical (mean 6.68 dB) for Enc-AFDM and standard AFDM; the CPP-inclusive transmit-block complementary cumulative distribution function (CCDF) is also measured and matches closely between the two waveforms (mean transmit-block PAPR ≈ 6.72 dB, 99th percentile ≈ 9.3 dB).*

This property is important for practical satellite transmitters, where power amplifier efficiency depends on PAPR characteristics.

The number of possible permutations is N!. For the phase mask with *b*-bit quantization per sample, the phase space has $2^{bN}$ possibilities. The total key space size is $\left|K\right|=N!\times 2^{bN}$. For N=64 and b=8 bits, this gives $64!\times 2^{512}\approx 10^{89}\times 10^{154}$. A large mask configuration space is necessary but not sufficient for security. The brute-force boundary is the entropy of the master key *K* driving HMAC-SM3. The combinatorial mask diversity $\left|K\right|=N!\times 2^{bN}$ says nothing about structural attacks (Section 4). The combinatorial mask diversity should therefore be read as the size of the configuration space reachable from the HMAC-SM3 expansion, not as a brute-force security guarantee (which is determined by the master-key entropy, set by the key agreement rather than by the mask configuration space).

Algorithm 2 summarizes the complete transmit and legitimate-receive chains. The effective channel ${\tilde{H}}_{\mathrm{eff}}$ used in step 4 of the receiver is obtained from the secret-pilot estimation scheme developed next.
**Algorithm 2** Encrypted-AFDM transmitter and decrypt-first receiver.
**Transmitter** (per frame *t*, key *K*)1:Derive R,A from HMAC-SM3(K) and pilot phases $\left\{\theta_{p}^{\left(t\right)}\right\}$ from HMAC-SM3(K,t)
2:Map bits to DAFT-domain data/pilot symbols x3:$s\leftarrow D^{-1}\left(x\right)=\Lambda_{c_{1}}^{H}F^{H}\Lambda_{c_{2}}^{H}x$4:$s_{\mathrm{enc}}\leftarrow ARs$▷ time-domain mask5:$s_{\mathrm{tx}}\leftarrow \mathrm{add}\text{\_}\mathrm{CPP}\left(s_{\mathrm{enc}}\right)$▷ chirp-periodic prefix6:transmit $s_{\mathrm{tx}}$
**Receiver** (Bob, key *K*)1:remove CPP: $r\leftarrow r_{\mathrm{rx}}\left[L_{\mathrm{cp}}:\;\right]$2:decrypt: $r_{\mathrm{dec}}\leftarrow R^{-1}A^{H}r$3:demodulate: $y_{\mathrm{dec}}\leftarrow D\left(r_{\mathrm{dec}}\right)$4:equalize: $\hat{x}\leftarrow {\left({\tilde{H}}_{\mathrm{eff}}^{H}{\tilde{H}}_{\mathrm{eff}}+\sigma^{2}\lambda I\right)}^{-1}{\tilde{H}}_{\mathrm{eff}}^{H}y_{\mathrm{dec}}$5:extract data subcarriers and QAM-demodulate

### 3.8. Encrypted Training Signal and Secure Channel Estimation

Channel estimation in AFDM is challenging because $H_{\mathrm{eff}}$ is not diagonal. It is a sparse shifted-diagonal matrix under integer Doppler (at most *P* nonzeros per row). The conventional approach of transmitting pilots on a subset of subcarriers and performing per-subcarrier least-squares (LS) estimation fails because the received symbol at DAFT position *q* comes from a different transmitted position *p*, determined by the path-dependent shift ${\mathrm{loc}}_{i}=\left(\alpha_{i}+2Nc_{1}l_{i}\right)\;\mathrm{mod}\;N$.

Encrypted-AFDM introduces an additional challenge. The encryption transforms the effective channel. To address both challenges simultaneously, we propose a full-band pilot-based estimation scheme with key-derived secret pilot phases.

Alice transmits *N* pilot symbols on the downlink, one per DAFT subcarrier. The pilot at subcarrier *p* is:

(16) $$x_{p}^{\mathrm{pilot}}=e^{j\theta_{p}},\;\theta_{p}=\mathrm{PRF}\left(K,p,\mathrm{frame}\text{\_}\mathrm{id}\right),$$

where PRF is a pseudo-random function (modeled via a cryptographic key derivation function in practice; instantiated via HMAC-SM3 key derivation in our experiments). The phases $\left\{\theta_{p}\right\}$ are known only to Alice and Bob. Concretely, the secret pilot phases are generated from the shared key *K* and a per-frame nonce *t* as

(17) $$\theta_{p}^{\left(t\right)}=\frac{2\pi }{2^{b}}\left(\mathrm{HMAC}\text{-}\mathrm{SM}3\;\left(K,\;\mathrm{AFDM}\text{-}\mathrm{ENC}\text{-}\mathrm{pilot}\;|\;\underline{t}\;|\;\underline{p}\right)\;\mathrm{mod}\;2^{b}\right),$$

where p∈{1,…,N} is the subcarrier index, *b* is the per-phase quantization (b=8 in our experiments), and $\underline{\cdot }$ denotes fixed-length big-endian encoding. All key-derived quantities are produced by a single domain-separated key-derivation function built on HMAC-SM3 (RFC 2104) [26], using fixed, distinct ASCII labels as is standard in HMAC-based key derivation. Writing $H_{K}\left(m\right)≜\mathrm{HMAC}\text{-}\mathrm{SM}3\left(K,m\right)$, with the 256-bit SM3 digest read in big-endian order, the permutation and phase mask use the labels AFDM-ENC-perm and AFDM-ENC-phase,

$$R\leftarrow \mathrm{FY}\;\left(H_{K}\left(\mathrm{AFDM}\text{-}\mathrm{ENC}\text{-}\mathrm{perm}\right)\right),\;\phi_{n}=\frac{2\pi }{2^{b}}\;{\mathrm{LSB}}_{b}\;\left(H_{K}\left(\mathrm{AFDM}\text{-}\mathrm{ENC}\text{-}\mathrm{phase}\;|\;\underline{n}\right)\right),\;A=\mathrm{diag}\left(e^{j\phi_{n}}\right),$$

where $\underline{\cdot }$ is fixed-width big-endian (integer-to-octet-string) encoding—$\left\lceil b_{t}/8\right\rceil$ bytes for the nonce *t*, two bytes for the subcarrier/sample indices n,p (sufficient for $N\leq 2^{16}$), and four bytes for the keystream counter *c*—and ${\mathrm{LSB}}_{b}\left(\cdot \right)$ selects the *b* least-significant bits, which equals reduction modulo $2^{b}$ and introduces no modulo bias. The permutation is a Fisher–Yates shuffle driven by the counter-mode keystream $Z_{c}=H_{K}\left(\mathrm{AFDM}\text{-}\mathrm{ENC}\text{-}\mathrm{perm}\;|\;\underline{c}\right)$, c=0,1,…, parsed as big-endian integers, with rejection sampling at each shuffle step so each swap index is uniform over its range. The three labels make the HMAC input domains disjoint; under the PRF assumption on HMAC-SM3 the three derivations are computationally domain-separated—two output values may still coincide, which does not indicate input reuse—and the fixed-width encoding of n,p,t prevents prefix-collision ambiguity such as (*p* = 1, *t* =11) versus (*p* = 11, *t* = 1).

We prevent nonce reuse by construction, without claiming that the quantized phase values are unique. The nonce *t* is a monotonically increasing per-frame counter, allocated and persisted atomically by the transmitter before each frame and never decremented, rolled back, or reused under the same *K* (packet loss may create counter gaps but never rollback). Distinct (t,p) pairs therefore yield distinct HMAC inputs; after reduction to *b* bits, however, equal phase values remain possible, and under the PRF model any two distinct inputs yield the same *b*-bit phase with probability $2^{-b}$, so security does not rely on uniqueness of the quantized phases. Counter uniqueness is scoped to each (K,sender) pair: if multiple transmitters or streams share *K*, they use independent keys or include a fixed-width sender identifier in the HMAC input. To rule out wrap-around reuse, a fresh, non-repeating master key is installed through the authenticated key agreement of the threat model before *t* reaches its $2^{b_{t}}$ ceiling, where $b_{t}$ is the counter width chosen so that $2^{b_{t}}$ frames exceed the key lifetime; if rekeying is unavailable, the transmitter fails closed. With $b_{t}=32$ and a 1 ms frame, $2^{b_{t}}$ frames span about 49.7 days, while $b_{t}=64$ makes exhaustion negligible over any practical lifetime. Under these state-management assumptions, the derivation (17) is free of nonce and input reuse. The per-frame nonce *t* makes the phases frame-dependent; Section 4.5 demonstrates this freshness is mandatory rather than optional. Our simulations against adversaries A1–A3 use a static key; the nonce-based freshness defense against the single-known-frame adversary (Section 4.5) is validated in Section 5.12, where per-frame nonces are shown to defeat the cross-frame anchor attack. Same-frame known plaintext remains the open boundary.

Bob receives the encrypted pilot transmission through the doubly dispersive channel. His estimation procedure is:

1. For each pilot subcarrier p=1,…,N, the transmitted signal is $s_{p}=D^{-1}\left(e^{j\theta_{p}}e_{p}\right)$, where $e_{p}$ is the *p*-th standard basis vector.
2. After encryption, CPP addition, channel propagation, CPP removal, and time-domain decryption, Bob computes the DAFT: $y_{p}^{\mathrm{dec}}=D\left({\mathrm{Dec}}_{K}\left(r_{p}^{\mathrm{rx}}\right)\right)$.
3. The *p*-th column of the estimated equivalent channel ${\tilde{H}}_{\mathrm{eff}}$ is:
  (18) $${\tilde{H}}_{\mathrm{eff}}\left[:,p\right]=y_{p}^{\mathrm{dec}}\;e^{-j\theta_{p}}.$$

The estimated channel ${\tilde{H}}_{\mathrm{eff}}$ models the equivalent DAFT-domain channel after decryption, i.e., ${\tilde{H}}_{\mathrm{eff}}=D\circ {\mathrm{Dec}}_{K}\circ H\circ {\mathrm{Enc}}_{K}\circ D^{-1}$, where H denotes the physical channel operator. After arbitrary permutation and phase rotation, ${\tilde{H}}_{\mathrm{eff}}$ is typically an effectively dense N×N matrix; the one-nonzero-per-row sparsity of the original AFDM channel does not survive the encryption transformations. Our numerical construction handles the fully dense case without relying on sparsity assumptions. Crucially, although the mask destroys the sparsity pattern, it cannot reduce the rank: ${\tilde{H}}_{\mathrm{eff}}$ remains full-rank, as we now prove.

**Proposition** **2.** *Let $H:C^{N}\;\to \;C^{N}$ denote the time-domain doubly dispersive channel operator after CPP removal, and let ${\tilde{H}}_{\mathrm{eff}}=D\;{\mathrm{Dec}}_{K}\;H\;{\mathrm{Enc}}_{K}\;D^{-1}$. Then ${\tilde{H}}_{\mathrm{eff}}$ is unitarily similar to H,*

(19) $${\tilde{H}}_{\mathrm{eff}}=Q\;H\;Q^{H},\;Q≜D\;{\mathrm{Dec}}_{K},$$

*so $\mathrm{rank}\left({\tilde{H}}_{\mathrm{eff}}\right)=\mathrm{rank}\left(H\right)$ and, in fact, the two share the same singular values. This holds independently of the key K. For any fixed path geometry ${\left\{\left(l_{i},\alpha_{i}\right)\right\}}_{i=1}^{P}$ there is a Lebesgue-null set $Z\subset C^{P}$, independent of the key K, such that H—and hence ${\tilde{H}}_{\mathrm{eff}}$—is nonsingular for every $\{h_{i}\}\notin Z$. Equivalently, nonsingularity holds almost surely whenever the path-gain vector has an absolutely continuous joint distribution (e.g., the Rayleigh model of Section 5). Thus, ${\tilde{H}}_{\mathrm{eff}}$ is full-rank for every valid key and almost every path-gain tuple at any fixed geometry.*

**Proof.**  **Rank preservation via unitary similarity.** Since ${\mathrm{Dec}}_{K}={\mathrm{Enc}}_{K}^{-1}={\left(AR\right)}^{-1}=R^{-1}A^{H}$, define $Q≜D\;{\mathrm{Dec}}_{K}$. Both $D=\Lambda_{c_{2}}F\Lambda_{c_{1}}$ (a product of the unitary DFT matrix F and two unit-modulus diagonal chirp matrices) and ${\mathrm{Dec}}_{K}$ are unitary, hence Q is unitary, and

$${\tilde{H}}_{\mathrm{eff}}=D\;{\mathrm{Dec}}_{K}\;H\;{\mathrm{Enc}}_{K}\;D^{-1}=\left(D\;{\mathrm{Dec}}_{K}\right)\;H\;{\left(D\;{\mathrm{Dec}}_{K}\right)}^{-1}=Q\;H\;Q^{H},$$

because ${\left(D\;{\mathrm{Dec}}_{K}\right)}^{-1}={\mathrm{Enc}}_{K}\;D^{-1}$. Unitary similarity preserves rank, determinant, eigenvalues, and singular values, so $\mathrm{rank}\left({\tilde{H}}_{\mathrm{eff}}\right)=\mathrm{rank}\left(H\right)$. The key enters only through Q and cannot alter the rank.

**Generic nonsingularity of H.** The block input–output relation (7) (with w=0) is the linear operator $H=\sum_{i=1}^{P}h_{i}\;U_{i}$, where $U_{i}=M_{\alpha_{i}}C_{l_{i}}$ first applies the chirp-periodic cyclic delay $C_{l_{i}}$ and then the Doppler modulation $M_{\alpha_{i}}$: $\left(U_{i}s\right)\left[n\right]=e^{j2\pi \alpha_{i}n/N}\;\left(C_{l_{i}}s\right)\left[n\right]$, with $\left(C_{l}s\right)\left[n\right]=\kappa_{l}\left[n\right]\;s\left[\left(n-l\right)\;\mathrm{mod}\;N\right]$, $\kappa_{l}\left[n\right]=1$ for n≥l and $\kappa_{l}\left[n\right]=e^{-j2\pi c_{1}\left(N^{2}+2N\left(n-l\right)\right)}$ for n<l the unit-modulus CPP wrap factor implied by (6). Each $U_{i}$ is unitary. The factor $M_{\alpha_{i}}=\mathrm{diag}\left(e^{j2\pi \alpha_{i}n/N}\right)$ is unitary for every real $\alpha_{i}$ (integer or fractional), and $C_{l_{i}}$ is monomial unitary (exactly one unit-modulus entry per row and column). Hence $|\det(U_{i})|=1$. For fixed geometry, $p\left(\left\{h_{i}\right\}\right)≜\det\left(H\right)$ is a homogeneous polynomial of degree *N* in $\left(h_{1},\ldots ,h_{P}\right)$. It is not the zero polynomial: the single-path specialization $h_{i^{*}}\neq 0$, $h_{i}=0$ for $i\neq i^{*}$, gives $H=h_{i^{*}}U_{i^{*}}$ with $\left|\det\left(H\right)|=\right|h_{i^{*}}{|}^{N}>0$. A nonzero polynomial in *P* complex variables vanishes only on a set of 2P-dimensional Lebesgue measure zero; hence det(H)≠0 for every $\{h_{i}\}\notin Z$ (a null, key-independent set), and by (19) ${\tilde{H}}_{\mathrm{eff}}$ is full-rank for every valid key and almost every path-gain tuple at the fixed geometry. □

**Remark** **3.** *In (19) the right factor is the inverse of the left, so the mask acts as a unitary change in basis: it cannot reduce the rank or alter the singular values of the time-domain channel. What it can change is the sparsity pattern. In the numerical realizations of this paper, the post-decryption channel is typically effectively dense, but this is an empirical observation, not a consequence of rank preservation (sparsity and rank being independent—a dense matrix may be singular and a sparse one full-rank). Two scope limits should be noted. The proposition gives no lower bound on the smallest singular value, so channels arbitrarily close to the exceptional (measure-zero) set may be ill-conditioned. The regularized LMMSE filter ${\tilde{H}}_{\mathrm{eff}}^{H}{\tilde{H}}_{\mathrm{eff}}+\sigma^{2}\lambda I$ is positive definite for any $\sigma^{2}\lambda >0$ irrespective of rank, so equalization is well-posed for reasons independent of this rank result.*

If pilots were public (e.g., $x_{p}^{\mathrm{pilot}}=1$ for all *p*), Eve could estimate the composite channel $G=D\circ H\circ {\mathrm{Enc}}_{K}\circ D^{-1}$ directly from the received pilot signals without knowing the key. Since the encryption is a linear transformation, it can be “absorbed” into the channel estimate: $G_{\mathrm{est}}\left[:,p\right]=y_{p}^{\mathrm{rx}}$, where $y_{p}^{\mathrm{rx}}$ is the raw DAFT-domain received pilot (no decryption). Eve could then use $G_{\mathrm{est}}$ in a standard LMMSE equalizer to decode subsequent data frames. Secret pilots block this attack because Eve receives $y_{p}^{\mathrm{rx}}=G\left[:,p\right]\;e^{j\theta_{p}}+n$, and without knowing $\theta_{p}$, she cannot separate the channel gain from the unknown phase. When Eve equalizes using the phase-corrupted estimate, each decoded symbol is multiplied by a random phase factor $e^{-j\theta_{p}}$, producing random constellation rotations and hence BER near 0.5 under the naive detector (possibly biased above or below 0.5 depending on the decision rule; Section 4.1 discusses the interpretation).

### 3.9. Decrypt-First Receiver Architecture

The legitimate receiver employs a decrypt-first architecture, which minimizes the equalization cost for the encrypted waveform:

1. CPP removal: Remove the first $L_{\mathrm{cp}}$ samples from the received signal.
2. Time-domain decryption: $r_{\mathrm{dec}}={\mathrm{Dec}}_{K}\left(r\right)=R^{-1}A^{H}r$.
3. DAFT demodulation: $y_{\mathrm{dec}}=D\left(r_{\mathrm{dec}}\right)$.
4. LMMSE equalization: Using the estimated equivalent channel ${\tilde{H}}_{\mathrm{eff}}$:
  (20) $$\hat{x}={\left({\tilde{H}}_{\mathrm{eff}}^{H}{\tilde{H}}_{\mathrm{eff}}+\sigma^{2}\lambda I\right)}^{-1}{\tilde{H}}_{\mathrm{eff}}^{H}y_{\mathrm{dec}},$$
  where λ=1 (standard LMMSE; the regularization matches the noise variance $\sigma^{2}$).
5. Symbol demodulation: Extract the data subcarriers (positions $\alpha_{\max}+1$ to $N-\alpha_{\max}$) and perform QAM demodulation.

This is in contrast to an alternative “equalize-first” architecture where DAFT demodulation and LMMSE equalization precede decryption. Because the time-domain decryption ${\mathrm{Dec}}_{K}$ is unitary and applied before the DAFT, the two orderings yield equivalent BER; the decrypt-first approach is computationally simpler (the DAFT-domain symbols are obtained directly after equalization, with no need for an additional IDAFT–decrypt–DAFT round-trip), so we adopt it throughout and do not separately benchmark the equalize-first variant.

We make the equivalence precise, since it justifies discarding the equalize-first branch without a separate experiment.

**Proposition** **3.** *Let $U=R^{-1}A^{H}$ denote the (unitary) time-domain decryption and let the DAFT be $D=\Lambda_{c_{2}}F\Lambda_{c_{1}}$. Consider two receivers operating on the same CPP-removed observation r: (a) decrypt-first, which forms $y_{\mathrm{dec}}=DUr$ and applies the LMMSE filter built from ${\tilde{H}}_{\mathrm{eff}}=DU\;H\;{\mathrm{Enc}}_{K}D^{-1}$; and (b) equalize-first, which forms y=Dr, applies the LMMSE filter built from the corresponding un-decrypted effective channel, and then removes the residual mask. If both filters use the MMSE regularization consistent with their respective effective channels, they produce identical soft estimates $\hat{x}$, and hence identical hard decisions and BER.*

**Proof.**  Both effective channels differ by left-multiplication by the fixed unitary $V≜DUD^{-1}$: writing the equalize-first effective channel as $H^{\prime }=D\;H\;{\mathrm{Enc}}_{K}D^{-1}$, one has ${\tilde{H}}_{\mathrm{eff}}=VH^{\prime }$ and $y_{\mathrm{dec}}=Vy$. The MMSE filter is covariant under a unitary change of observation basis. For any unitary V, ${\left({\left(H^{\prime }\right)}^{H}V^{H}VH^{\prime }+\sigma^{2}\lambda I\right)}^{-1}{\left(H^{\prime }\right)}^{H}V^{H}\left(Vy\right)={\left({\left(H^{\prime }\right)}^{H}H^{\prime }+\sigma^{2}\lambda I\right)}^{-1}{\left(H^{\prime }\right)}^{H}y$, since $V^{H}V=I$. The two soft outputs therefore coincide. □

The practical import of Proposition 3 is that the choice between the two architectures is purely one of implementation cost, and the cheaper decrypt-first ordering loses nothing in performance. It also clarifies why the mask is invisible to Bob’s error rate: for the legitimate key, U exactly inverts ${\mathrm{Enc}}_{K}$, so the two receiver architectures produce identical estimates by Proposition 3. The effective channel ${\tilde{H}}_{\mathrm{eff}}$ seen by the decrypt-first receiver differs from the original AFDM effective channel $H_{\mathrm{eff}}$ through the unitary similarity transformation induced by the mask; numerical evidence (Section 5, legitimate-link benchmark) confirms the BER penalty is below 0.5 dB.

### 3.10. Estimation-Error Propagation and Regularization

The LMMSE equalizer (20) uses the secret-pilot estimate ${\tilde{H}}_{\mathrm{eff}}$ rather than the true effective channel. We characterize the pilot-estimation residual and the role of the time-domain decryption.

Let ${\tilde{H}}_{\mathrm{eff}}^{★}$ be the true post-decryption effective channel and let each pilot be the energy-$E_{p}$ symbol $\sqrt{E_{p}}\;e^{j\theta_{p}}e_{p}$ on a single DAFT subcarrier (the unit-modulus pilot of (16) corresponds to $E_{p}=1$). Assuming the channel is constant over the *N* pilot blocks and the pilot noises are independent, the least-squares estimate ${\tilde{H}}_{\mathrm{eff}}\left[:,p\right]=y_{p}^{\mathrm{dec}}\;e^{-j\theta_{p}}/\sqrt{E_{p}}$ satisfies ${\tilde{H}}_{\mathrm{eff}}={\tilde{H}}_{\mathrm{eff}}^{★}+E$ with $E\left[:,p\right]={\tilde{n}}_{p}\;e^{-j\theta_{p}}/\sqrt{E_{p}}$, where ${\tilde{n}}_{p}=D\;{\mathrm{Dec}}_{K}\;w_{p}$ is the DAFT-domain pilot noise and $\theta_{p}$ the key-derived phase (17). The pilot phase does not itself create a residual: Alice and Bob evaluate the same deterministic *b*-bit quantizer on the shared key—for instance $\theta_{p}=2\pi \;\left(\mathrm{HMAC}\left(K,p,t\right)\;\mathrm{mod}\;2^{b}\right)/2^{b}$—so the de-rotation $e^{-j\theta_{p}}$ is exact, and the unit-modulus factor only rotates the noise. (Residuals from oscillator phase noise, residual carrier frequency offset (CFO), or nonce/index disagreement are synchronization/implementation errors, distinct from *b*-bit phase quantization, and are excluded here.)

**Lemma** **1.** *Under the above assumptions, $\mathrm{Cov}\left(\mathrm{vec}E\right)=\left(\sigma^{2}/E_{p}\right)\;I_{N^{2}}$, independently of the key K and of the phase quantization. The decryption ${\mathrm{Dec}}_{K}$ is a unitary change in the noise basis. It preserves the Frobenius norm of the residual and, as pilot noise is white, its covariance.*

**Proof.**  With $U≜D\;{\mathrm{Dec}}_{K}$ unitary and $w_{p}∼\mathrm{CN}\left(0,\sigma^{2}I\right)$, the DAFT-domain pilot noise has $\mathrm{Cov}\left({\tilde{n}}_{p}\right)=U\;\sigma^{2}I\;U^{H}=\sigma^{2}I$. Scaling by $1/\sqrt{E_{p}}$ and the unit-modulus rotation then give $\mathrm{Cov}\left(E\left[:,p\right]\right)=\left(\sigma^{2}/E_{p}\right)I$, and independence across the *N* pilots yields $\mathrm{Cov}\left(\mathrm{vec}E\right)=\left(\sigma^{2}/E_{p}\right)I_{N^{2}}$, the covariance of an unencrypted one-hot columnwise least-squares estimator with the same pilot energy. Decryption changes only the noise basis, not its strength. □

Lemma 1 concerns the residual E, not the post-equalizer MSE. With $W={\left({\tilde{H}}_{\mathrm{eff}}^{H}{\tilde{H}}_{\mathrm{eff}}+\sigma^{2}\lambda I\right)}^{-1}{\tilde{H}}_{\mathrm{eff}}^{H}$ and $y_{\mathrm{dec}}={\tilde{H}}_{\mathrm{eff}}^{★}x+\tilde{n}$, $\hat{x}-x=\left(W{\tilde{H}}_{\mathrm{eff}}^{★}-I\right)x+W\tilde{n}$. Using ${\tilde{H}}_{\mathrm{eff}}^{★}={\tilde{H}}_{\mathrm{eff}}-E$, the channel-estimation-induced contribution obeys, for data x on the $N_{d}$ active subcarriers, $E_{x}{∥WEx∥}^{2}\leq {∥W∥}_{2}^{2}\;E_{x}{∥Ex∥}^{2}$, with the universal ridge bound ${∥W∥}_{2}=\max_{i}s_{i}/\left(s_{i}^{2}+\sigma^{2}\lambda \right)\leq {\left(2\sqrt{\sigma^{2}\lambda }\right)}^{-1}$, where $\left\{s_{i}\right\}$ are the singular values of the estimate ${\tilde{H}}_{\mathrm{eff}}$. By Lemma 1 the statistics of E are decryption-invariant, while for every fixed λ>0 the bound on ${∥W∥}_{2}$ is uniform over the estimated-channel singular values. The pilot-to-genie BER gap observed in Section 5 reflects this total channel-estimation degradation.

Under perfect CSI and isotropic unit-power symbols, the linear-MMSE-optimal regularization matches the noise variance, $\lambda^{*}=1$, regardless of channel sparsity (sparsity lowers equalization complexity but not the linear-MMSE regularization). By Proposition 2, the singular values of the true channel—and hence this optimum—are unchanged by decryption. Under pilot estimation, a common i.i.d.-error effective-noise heuristic suggests $\lambda_{\mathrm{eff}}\approx 1+N_{d}\;\sigma_{E}^{2}/\sigma^{2}$ (with $\sigma_{E}^{2}=\sigma^{2}/E_{p}$ and $N_{d}$ active data subcarriers). This is not the exact optimizer, however, because E and the estimate ${\tilde{H}}_{\mathrm{eff}}={\tilde{H}}_{\mathrm{eff}}^{★}+E$ are statistically coupled. The value λ=1 is the linear-MMSE optimum under the isotropic model and the regularization adopted for the numerically known-channel benchmark of Section 5. For the pilot-estimated link, it is a simple, fixed choice, and tuning λ is a straightforward refinement.

### 3.11. Computational Complexity

Table 4 summarizes the per-block computational complexity. The IDAFT costs O(NlogN)+2N, identical to plain AFDM. The time-domain mask adds O(N) work (permutation plus *N* phase multiplications), and HMAC-SM3 key derivation is O(N). The dominant cost at the legitimate receiver is the dense LMMSE equalizer at $O(N^{3})$, versus O(N) for standard AFDM under integer Doppler; this cost is amortizable across *D* data blocks of a coherence interval (per-block $O(N^{2}+N^{3}/D)$, approaching $O(N^{2})$ only for a sufficiently long reuse interval). A passive adversary faces $O(N^{3}+KN)$ per attack window for composite channel estimation plus blind phase recovery. The security does not arise from imposing a super-polynomial workload on Eve, but from the per-frame nonce denying cross-frame validity (Section 4.5).

### 3.12. Training Overhead and Spectral Efficiency

One channel estimate requires *N* separate block transmissions (one pilot per DAFT subcarrier). With *D* data blocks per training cycle and $N_{d}=N-2\alpha_{\max}$ active subcarriers, the spectral efficiency is $\eta =DN_{d}\log_{2}M/\left[\left(N+D\right)\left(N+L_{\mathrm{cp}}\right)\right]$ bpcu. Representative values range from η≈0.24 bpcu (*D* = 10, *N* = 64, QPSK) to η≈2.29 bpcu (*D* = 200, *N* = 128, 16QAM); the overhead is governed by D/N and is competitive only when the channel is stable over many blocks. Reducing training cost through compressed or semi-blind estimation is an important future direction (Section 6).

## 4. Security Analysis

### 4.1. Security Objective and Adversary Classes

We state the security objective precisely to avoid overclaiming. We do not claim information-theoretic secrecy or a reduction to a hard cryptographic problem. Instead, the target is computational obfuscation against passive receivers. For an eavesdropper drawn from a specified class A, the decoded data should be statistically close to a random guess, i.e., uncoded BER indistinguishable from that of an uninformed receiver. We consider four adversary classes of increasing strength. Class A1 is a wrong-key eavesdropper that applies the legitimate decrypt-then-LMMSE chain with an incorrect key. Class A2 is a composite-channel estimator that observes secret pilots and inverts the estimated linear map without decryption. Class A3 is a multi-frame known-plaintext attacker that performs naive least-squares recovery of the composite channel from data frames alone. Class A4 is a stronger passive adversary that combines the always-available composite estimate GΘ (from the secret pilots) with blind Viterbi–Viterbi phase recovery on the residual per-subcarrier rotation, optionally aided by a single known-plaintext frame. Our experiments evaluate A1–A4 (Section 4.5 and Section 5). Even so, our reported BER figures are upper bounds on the protection, not lower bounds on adversary error. Forward error correction (FEC)-aided and decision-directed joint attacks are characterized analytically (Section 4.5) and validated with a concrete LDPC code (Section 5.17).

We also caution on interpretation. The meaningful obfuscation target is BER indistinguishable from 0.5 (for a binary-equivalent metric). A receiver whose BER is consistently above 0.5 is not “more secure”; such a bias is itself information an adversary can exploit by flipping or de-rotating decisions. Where our experiments report values slightly above 0.5, this reflects a specific naive receiver under structured phase interference, not additional security margin.

We formalize the target as an obfuscation game, deliberately weaker than a cryptographic indistinguishability game, so that our claims match the evidence we provide.

**Definition** **2.** *Let A be a class of passive receivers (adversary strategies) with the capabilities enumerated in the threat model. The Encrypted-AFDM waveform achieves (A,δ)-obfuscation at a given operating point if, for every adversary A∈A and a key drawn uniformly from K, the expected uncoded BER satisfies $|\;E[{\mathrm{BER}}_{A}]-0.5\;|\leq \delta$, where the expectation is over the key, data, channel, and noise. Smaller δ (closer to a random guess) is stronger.*

Definition 2 makes three limitations explicit. First, it is stated relative to a class A: a scheme can be (A,δ)-obfuscating for a weak class and fail badly for a stronger one, which is exactly what we observe when we move from A1 to the single-known-frame variant of A4. Second, it uses uncoded BER, a proxy that ignores soft information and coding gain available to a real adversary. Third, it is an empirical statement verified by simulation at specific operating points, not a proof over all inputs. Table 5 summarizes the four classes and the experiment that evaluates each.

### 4.2. Wrong-Key Decoding Degradation and Brute-Force Resistance

When Eve attempts to decode with a wrong key $K^{\prime }\neq K$, she applies ${\mathrm{Dec}}_{K^{\prime }}\left(r\right)={R^{\prime }}^{-1}{\left(A^{\prime }\right)}^{H}r$. Since ${R^{\prime }}^{-1}R\neq I$ (different permutation) and ${\left(A^{\prime }\right)}^{H}A\neq I$ (different phase mask), the decrypted signal is:

(21) $$r_{\mathrm{eve}}={R^{\prime }}^{-1}{\left(A^{\prime }\right)}^{H}ARs+{\tilde{n}}_{\mathrm{eve}},$$

where ${\tilde{n}}_{\mathrm{eve}}$ is the transformed noise. The composite matrix $C≜{R^{\prime }}^{-1}{\left(A^{\prime }\right)}^{H}AR$ is a monomial (generalized-permutation) matrix: exactly one nonzero, unit-modulus entry per row, determined by the mismatch between the true and guessed keys: the phase-mask mismatch ${\left(A^{\prime }\right)}^{H}A$ contributes an independent phase $e^{j(\phi_{n}-\phi_{n}^{\prime })}$ to each sample, and the permutation mismatch ${R^{\prime }}^{-1}R$ relocates samples according to a random permutation. Although C acts sample-wise in the time domain, Eve subsequently applies the DAFT to these permuted, phase-scrambled samples; the DAFT of a randomly permuted, randomly phase-rotated sequence spreads each transmitted DAFT-domain symbol’s energy across many output subcarriers with pseudo-random phases, so the recovered symbol at any subcarrier is a superposition of many independent random-phase contributions. By a central-limit heuristic, the equalizer output is then approximately circularly symmetric and statistically uncorrelated with the transmitted constellation, driving the QPSK hard-decision BER to 0.5. As confirmed by our simulations (Section 5), a wrong-key Eve employing a naive LMMSE receiver achieves uncoded BER near 0.5 across all SNRs for QPSK modulation. BER near 0.5 under a specific receiver does not constitute a security proof; more sophisticated receivers exploiting constellation structure, forward error correction, or decision-directed processing may achieve lower effective error rates.

### 4.3. Composite-Channel Estimation and Secret Pilot Defense

A more sophisticated passive attack is the composite-channel estimation attack. Eve receives the obfuscated pilot transmissions and attempts to estimate the composite linear map $G=D\circ H\circ {\mathrm{Enc}}_{K}\circ D^{-1}$ directly, without knowing the key.

For each pilot subcarrier *p*, Alice transmits a one-hot DAFT vector $e_{p}$ scaled by the secret phase $\theta_{p}$. Eve receives (after CPP removal and DAFT, without decryption):

(22) $$y_{p}^{\mathrm{rx}}=G\;e_{p}\;e^{j\theta_{p}}+\tilde{n}=G\left[:,p\right]\;e^{j\theta_{p}}+\tilde{n},$$

where $\theta_{p}$ is the (unknown) secret pilot phase. If pilots were public ($\theta_{p}=0$, i.e., $e^{j\theta_{p}}=1$), Eve could directly estimate $G\left[:,p\right]=y_{p}^{\mathrm{rx}}$ and construct the full composite channel from *N* pilot measurements. She could then equalize data frames via $\hat{x}=G_{\mathrm{est}}^{-1}y_{\mathrm{data}}$ without ever knowing *K*.

When $\theta_{p}$ are secret and key-derived, Eve’s estimate becomes $G_{\mathrm{est}}\left[:,p\right]=G\left[:,p\right]\;e^{j\theta_{p}}+\tilde{n}$. Defining $\Theta =\mathrm{diag}(e^{j\theta_{1}},\ldots ,e^{j\theta_{N}})$, we have $G_{\mathrm{est}}=G\Theta +\mathrm{noise}$. For data equalization:

(23) $${\hat{x}}_{\mathrm{eve}}=G_{\mathrm{est}}^{-1}y_{\mathrm{data}}\approx \Theta^{-1}G^{-1}Gx=\Theta^{-1}x.$$

Each symbol is multiplied by the inverse of an unknown random phase: ${\hat{x}}_{\mathrm{eve}}\left[p\right]=x\left[p\right]\;e^{-j\theta_{p}}$. For QPSK, where the constellation points lie at $\left\{\pm 1\pm j\right\}/\sqrt{2}$, the multiplication by a random unit-modulus complex number maps each point to a uniformly random point on the unit circle. The resulting BER approaches 0.5 (for QPSK) or exceeds it due to structured interference when the phase rotation maps a QPSK point near the decision boundary of another constellation point.

### 4.4. Known-Plaintext Attack Resistance

A more powerful adversary may have access to known plaintext symbols (for instance, from standardized frame headers, synchronization sequences, or higher-layer protocol fields). We analyze two variants:

Eve knows a subset $P_{\mathrm{known}}\subset \left\{1,\ldots ,N_{d}\right\}$ of the data symbols, with $|P_{\mathrm{known}}|=N_{k}$. Since $H_{\mathrm{eff}}$ is not diagonal, knowledge of symbol x[p] does not directly reveal the channel gain at DAFT position *p*. A per-subcarrier interpolation attack (estimating gains at known positions and interpolating to unknown positions) is structurally frustrated because the effective channel is a permutation-like matrix: the received signal at subcarrier *q* depends on the transmitted symbol at a different subcarrier p≠q, so knowledge of x[p] does not localize a channel coefficient at position *p*. We present this as an analytical argument only; we do not evaluate a single-frame partial-plaintext attack experimentally, and quantifying it (together with the stronger adversaries of Section 4.5) is left to future work.

If Eve can collect *M* frames where all *N* DAFT-domain symbols are known (e.g., from repeated known preambles), she can attempt least-squares recovery of the composite channel. For each frame m=1,…,M she records the DAFT-domain received vector $y_{m}$ and the known transmitted DAFT vector $x_{m}$, and stacks them into $Y=\left[y_{1},\ldots ,y_{M}\right]\in C^{N\times M}$ and $X=\left[x_{1},\ldots ,x_{M}\right]\in C^{N\times M}$. The LS estimate of the composite channel G (which folds in both the physical channel and Bob’s encryption) is:

(24) $$G_{\mathrm{LS}}=YX^{H}{\left(XX^{H}+\epsilon I\right)}^{-1},$$

where ϵ=0.01 provides Tikhonov regularization against ill-conditioning of $XX^{H}$. With M<N frames, $XX^{H}$ has rank min(M,N)=M, so $G_{\mathrm{LS}}$ captures only an *M*-dimensional subspace of the true *N*-dimensional channel. As *M* increases, Eve’s BER on subsequent frames decreases steadily. At M=N, with *N* linearly independent known frame vectors, X is full-rank and $G_{\mathrm{LS}}$ converges to the composite channel $G=D\circ H\circ {\mathrm{Enc}}_{K}\circ D^{-1}$ directly. Our experiments quantify this degradation: Eve’s BER is 0.458, 0.416, 0.321, and 0.106 for M=1,4,16,64, respectively, at 20 dB SNR with N=64. Note that the BER is already well below 0.5 at M=4 and M=16, so protection erodes gradually rather than holding until an M=N cliff.

Leakage grows steadily with *M*, and the key must be refreshed well before *N* fully known frames accumulate. This A3 characterization is superseded by the stronger A4 adversary (Section 4.5), which exploits the always-available composite estimate GΘ and shows that a single known-plaintext frame resolves the residual phase ambiguity and breaks composite-channel confidentiality; the operative requirement is therefore per-frame nonce-derived pilot phases.

Key reuse across frames is a related concern: if the same key is used for two frames, Eve’s naive composite estimate from Frame 1 applied to Frame 2 yields BER≈0.46–0.48 (phase-rotated, just below 0.5), but this does not establish resistance to joint multi-frame exploitation. The mandatory pilot-phase freshness (nonce) requirement from the stronger-adversary experiment (Section 4.5) governs both the KPA and key-reuse attack surfaces.

### 4.5. Blind Phase Recovery and Other Advanced Attacks

The secret pilot defense reduces the composite-channel attack to a per-subcarrier unknown phase ambiguity: Eve obtains ${\hat{x}}_{\mathrm{eve}}=\Theta^{-1}x$. This is not a security proof. Several techniques could resolve the unknown phases—blind search over quantized rotations, multi-frame clustering, known-header exploitation, decision-directed re-estimation, and FEC/CRC-aided hypothesis search—but rather than defer all of these, we evaluate the two most immediately available (blind Viterbi–Viterbi fourth-power phase recovery and a single known-plaintext anchor) as adversary class A4 (Section 5).

We first record the blind estimator precisely, since its structure explains why it fails under QPSK. On a given subcarrier, after the composite estimate GΘ is applied, Eve observes $z=e^{-j\theta }\;x+\mathrm{noise}$ with unknown rotation θ and *x* drawn from the QPSK alphabet ${\left\{e^{j(\pi /4+k\pi /2)}\right\}}_{k=0}^{3}$. The Viterbi–Viterbi estimator raises the observation to the fourth power to strip the data modulation (since $x^{4}=e^{j\pi }=-1$ is constant over the QPSK alphabet) and averages over *K* frames:

(25) $$\hat{\theta }=-\frac{1}{4}\;∠\;\left(\frac{1}{K}\sum_{k=1}^{K}z_{k}^{4}\right)+\frac{\pi }{4}.$$

Because the fourth power maps all four QPSK points to the same value, the estimate is only determined modulo π/2: any of the four rotations $\hat{\theta }+m\pi /2$, m∈{0,1,2,3}, is equally consistent with the observations. This four-fold ambiguity is exactly the QPSK rotational symmetry and cannot be resolved by any amount of blind averaging (larger *K* only sharpens the estimate within one π/2 sector). It is resolved by a single known symbol on that subcarrier, which fixes *m*; with full-band known plaintext, all *N* ambiguities are fixed at once. This is the analytical basis for the two empirical findings below.

The findings are twofold.

First (favorable): a purely blind adversary who observes even K=32 same-key frames and applies per-subcarrier fourth-power phase estimation via (25) cannot resolve the rotation. Because a QPSK constellation has four-fold rotational symmetry, the estimate is ambiguous modulo π/2, and Eve’s BER stays at 0.46–0.48 across all SNRs (a small bias below 0.5), no better than the naive composite receiver. The secret-pilot defense withstands blind phase recovery under QPSK in the tested setting.

Second, and more importantly: a single known-plaintext frame suffices to resolve the per-subcarrier ambiguity completely, after which Eve’s BER collapses with SNR (from 0.41 at 0 dB to $1.1\times 10^{-1}$ at 10 dB and $4.2\times 10^{-3}$ at 20 dB). This is a far tighter boundary than the naive multi-frame LS attack of Section 4.4, which needs *N* known frames: an adversary who exploits the received pilots to form GΘ need only resolve the *N* residual phases, for which one fully known frame is enough. Consequently, per-frame nonce-derived pilot phases are a requirement, not an optional hardening step (validated against the cross-frame anchor in Section 5.12). Without them, any single known-plaintext frame (e.g., a standardized preamble) breaks composite-channel confidentiality. This supersedes the *N*-frame guideline of the multi-frame KPA (Section 4.4) as the operative security requirement. We characterize the decision-directed and FEC-aided joint attacks next. Under per-frame nonces, they are not identifiable by a phase-identifiability failure (analytically, under the stated constant-modulus, single-observation assumptions; resistance against an implemented coded decoder is not evaluated), and under reused pilot phases they reduce to the rotational-symmetry case above. Per-frame nonce-derived pilots prevent the resolved phase from transferring across frames, but do not protect a single frame that itself contains known symbols on enough subcarriers (e.g., standardized headers or synchronization fields) to fix those phases within that frame. This same-frame known-plaintext boundary is the key open question for a stronger adversary model.

A natural concern is whether an outer channel code with soft-information decoding lets Eve resolve the per-subcarrier phase ambiguity without a known-plaintext frame. The answer depends on how many observations share each phase $\theta_{p}$, and it separates the reused-phase and per-frame-nonce settings.

When the pilot phases are reused without a nonce, the phase $\theta_{p}$ is constant across frames, so over *K* observed frames Eve has *K* samples sharing each $\theta_{p}$. A decision-directed or soft-decision receiver can then estimate $\theta_{p}$ iteratively from these repeated observations, and a strong outer code (low-density parity-check (LDPC), Polar, or Turbo) may lower the signal-to-noise ratio at which the preliminary symbol decisions are reliable enough for the loop to lock. The uncoded modulation-only estimator retains a per-subcarrier π/2 ambiguity for QPSK. A non-rotation-invariant code could in principle select among the quadrant assignments given enough reused-phase frames, but a single known frame fixes every subcarrier regardless. The operative defense is therefore the per-frame nonce.

Under a per-frame nonce, each $\theta_{p}$ is fresh and is observed only once, through a single noisy symbol $z_{p}=e^{-j\theta_{p}}x_{p}+n_{p}$. For constant-modulus signaling such as phase-shift keying (PSK), the phase is then not identifiable from the data. For any candidate symbol $c_{p}$ there is a phase $\theta_{p}^{\prime }$ that makes $e^{-j\theta_{p}^{\prime }}c_{p}$ coincide with the observation, so every codeword is consistent with some phase assignment. Equivalently, the likelihood of $z_{p}$ marginalized over the uniform unknown phase depends only on $|z_{p}|$ and is the same for every unit-modulus symbol, so soft-information decoding provides no information about the phases. A strong code constrains the data symbols but not the independent per-subcarrier rotations, so a coded or decision-directed adversary is not identifiable under the per-frame nonce in the constant-modulus case (validated with an LDPC code in Section 5.17). With amplitude-varying modulation such as 16QAM, the marginalized likelihood retains amplitude information, and this identifiability result no longer applies. This identifiability failure in the constant-modulus case is the reason nonces are mandatory rather than optional.

The same-frame known-plaintext boundary remains. A single frame that carries known symbols on the subcarriers whose data Eve wants fixes those phases directly, since a known $x_{p}$ yields the phase estimate ${\hat{\theta }}_{p}=-\arg\left(z_{p}x_{p}^{★}\right)$, whereas a cyclic-redundancy-check syndrome alone cannot identify the independent per-subcarrier rotations. Once Eve has formed the composite estimate, a known symbol on a subcarrier is observationally equivalent to a phase reference on that subcarrier, which is the channel-coding instance of the linear-cipher boundary of Section 6. We accordingly position time-domain obfuscation as a complement to upper-layer encryption, which handles same-frame known-plaintext and coded-adversary confidentiality.

We note a scope limitation of the current implementation. The sample mask R and phase mask A are derived once from the key and held static within a run; only the pilot phases are nonce-derived. Because R,A (and therefore the composite channel G) are static across frames, an adversary who recovers G directly from a single known data frame can equalize subsequent data frames regardless of the pilot-phase nonce. The per-frame nonce defense validated in Section 5.12 therefore blocks specifically the pilot-phase-transfer (cross-frame anchor) attack, in which Eve reuses the pilot-derived GΘ estimate on a later frame whose nonce differs. Full cross-frame data confidentiality would require refreshing the data mask R,A per frame, which is a straightforward extension of the same key-derivation function but is not evaluated in the present experiments.

This analysis also addresses the active-adversary gap noted by reviewers. The proposed scheme does not provide active integrity, authentication, replay protection, or availability guarantees. It provides data confidentiality against the passive adversary classes A1–A4 defined above. An active adversary who perturbs the doubly dispersive channel—through pilot jamming or timing manipulation—can degrade Bob’s channel estimation quality and thereby deny service. The mask generation is independent of the channel estimate, so active channel perturbation does not directly reveal the mask or the data symbols. However, we do not establish confidentiality against adaptive active attacks that observe receiver feedback, decryption behavior, or retransmissions, and such attacks remain outside the scope of this paper. Pilot authentication, jamming-resistant training, and frequency-hopping pilots are orthogonal countermeasures that can be layered on top but require separate analysis.

### 4.6. Sensing Preservation Under Encryption

AFDM is a dual-function waveform: each physical scatterer with delay $l_{i}$ and Doppler $\alpha_{i}$ maps to a localized response in the DAFT-domain effective channel $H_{\mathrm{eff}}$, whose sparse delay–Doppler support encodes the target scattering map and supports range/velocity estimation via the matched-filter ambiguity function. This is the sensing readout exploited in AFDM-ISAC systems [10].

Because the time-domain mask AR is a monomial (unitary) matrix, the key-holding receiver inverts it exactly; the post-decryption effective channel ${\tilde{H}}_{\mathrm{eff}}$ is unitarily similar to the physical channel and preserves the delay–Doppler energy distribution of the standard AFDM channel for the evaluated geometry. Its matched-filter map concentrates energy at the true target cells, so sensing is preserved for the key holder under the evaluated channel model.

A receiver without the key applies standard AFDM processing to the encrypted waveform and observes the composite channel

(26) $$G=H_{\mathrm{eff}}P,\;P=DMD^{H},$$

where D is the DAFT matrix and M=AR is the time-domain mask. The delay–Doppler ambiguity function under the composite channel follows from the structure of P. For a single target at location $\left(l_{i},\alpha_{i}\right)$, the unencrypted effective channel has nonzero entries only along the shifted diagonal at offset ${\mathrm{loc}}_{i}=\left(2Nc_{1}l_{i}-\alpha_{i}\right)\;\mathrm{mod}\;N$. The no-key receiver’s ambiguity function for a candidate template at offset loc is

(27) $$\chi_{G}\left(\mathrm{loc}\right)=\sum_{p=0}^{N-1}|{\left[G\right]}_{p,(p+\mathrm{loc})\;\mathrm{mod}\;N}{|}^{2}=\sum_{p=0}^{N-1}|\sum_{q=0}^{N-1}{\left[H_{\mathrm{eff}}\right]}_{p,q}\;{\left[P\right]}_{q,(p+\mathrm{loc})\;\mathrm{mod}\;N}{|}^{2}.$$

Because P is a dense unitary matrix, the inner sum over *q* mixes all *N* columns of $H_{\mathrm{eff}}$ into every output entry. The energy that was concentrated along a single shifted diagonal in $H_{\mathrm{eff}}$ is redistributed across all diagonals of G, so the ambiguity function $\chi_{G}\left(\mathrm{loc}\right)$ has no sharp peak at the true target offset for the tested key realization. The matched-filter response against any single-target template is spread over all candidate cells, destroying target localization.

Section 5.13 shows the delay–Doppler matched-filter power maps for a four-scatterer channel (N=64, delays l∈{0,2,4,5}, Dopplers α∈{0,2,−1,3}, PDP [0,−3.6,−7.2,−10.8] dB). For the key holder, all matched-filter energy concentrates at the true target cells (sensing concentration =0 dB, with 2 of 4 targets exceeding the −6 dB detection threshold, consistent with the two strongest paths). For the no-key receiver, the energy is spread uniformly (sensing concentration =−16.6 dB, with 0 of 4 targets detected). This sensing-denial property follows from the specific monomial-mask structure of M and requires no modification to the encryption design. A noisy detection evaluation is reported in Section 5.13.

## 5. Results

### 5.1. Experimental Setup

We implemented Encrypted-AFDM in MATLAB R2026a (MathWorks, Inc., Natick, MA, USA) with the following default parameters unless otherwise specified: N=64 subcarriers, $\alpha_{\max}=1$, $l_{\max}=6$, $L_{\mathrm{cp}}=6$, QPSK modulation, 200–2000 Monte Carlo trials per SNR point (5× the prior budget, to smooth the high-SNR tail), SNR range 0 to 20 dB in 0.5 dB steps. The channel model is a 6-tap doubly dispersive Rayleigh fading channel with path losses [0,−3.6,−7.2,−10.8,−18.0,−25.2] dB and integer Doppler shifts uniformly distributed in $\left[-\alpha_{\max},\alpha_{\max}\right]$. The AFDM chirp parameters are $c_{1}=\left(2\alpha_{\max}+1\right)/\left(2N\right)$ and $c_{2}=\sqrt{2}/N$. Channel estimation uses *N* full-band secret pilot symbols per estimate. LMMSE equalization uses regularization λ=1 (standard LMMSE, with the regularization matching the noise variance $\sigma^{2}$). Encryption keys are 256-bit master keys processed via HMAC-SM3 (RFC 2104). Table 6 collects the complete parameter set used across all experiment blocks.

We evaluate fourteen experiment blocks covering eavesdropper BER, legitimate-link benchmark (including *N* = 128 with fractional Doppler), encrypted-pilot normalized mean-square error (NMSE), stage ablation, PAPR, symbol correlation, multi-frame known-plaintext attack, key-reuse attack, a stronger passive adversary with blind phase recovery, per-frame nonce-defense validation, and waveform-structure obfuscation.

### 5.2. Methodology and Statistical Treatment

Independent channel realizations and data frames are drawn per trial, and reported BERs are pooled over all trials and bits. To make the statistical treatment uniform across every experiment block and attack configuration, every BER block is treated under the same method: two-sided 95% Wilson score intervals on the pooled bit count, with the one-sided 95% Wilson upper bound at zero-error points; the figures plot pooled point estimates for clarity, while representative intervals for the security-critical configurations are reported in the text and summarized in Table 7. The pooled-bit Wilson intervals are nominal intervals under a bit-independence assumption; positive within-frame error correlation would reduce the effective sample size, so such intervals may under-cover (be too narrow) at the frame level, and a frame-clustered treatment would be more conservative. For the wrong-key Eve block, a frame-clustered bootstrap (10,000 resamples over 2000 independent frames) gives a 95% CI width within 1% of the pooled-bit Wilson interval, confirming that the pooled-bit intervals are adequate for the main security results. The obfuscation target is BER statistically indistinguishable from 0.5 (Section 4.1). An interval containing 0.5 indicates security under the tested receiver, while a bias above 0.5 is flagged as exploitable structure.

Trial budgets differ across blocks and are stated explicitly so that every interval is calibrated consistently. The legitimate-link and wrong-key BER blocks (E1, E2, E2b) use the full budget of 2000 frames/point (about $2.5\times 10^{5}$ bits). The attack blocks replay many known frames per point and therefore use smaller frame budgets: the multi-frame known-plaintext block (E7) uses an *M*-dependent budget of about 200 frames at *M* = 1 down to about 10 at *M* = 64, and the key-reuse (E8), stronger-adversary (E9), and nonce-defense (E10) blocks use about 40–50 frames/point (∼4–6 kbits). The Wilson intervals are computed at each block’s actual budget, so the attack-block intervals are wider than the legitimate-link intervals; we report them explicitly rather than omitting them, and we interpret the attack results as trends whose sign and order of magnitude are robust to the interval width.

### 5.3. Eavesdropper BER

**Figure 3:**
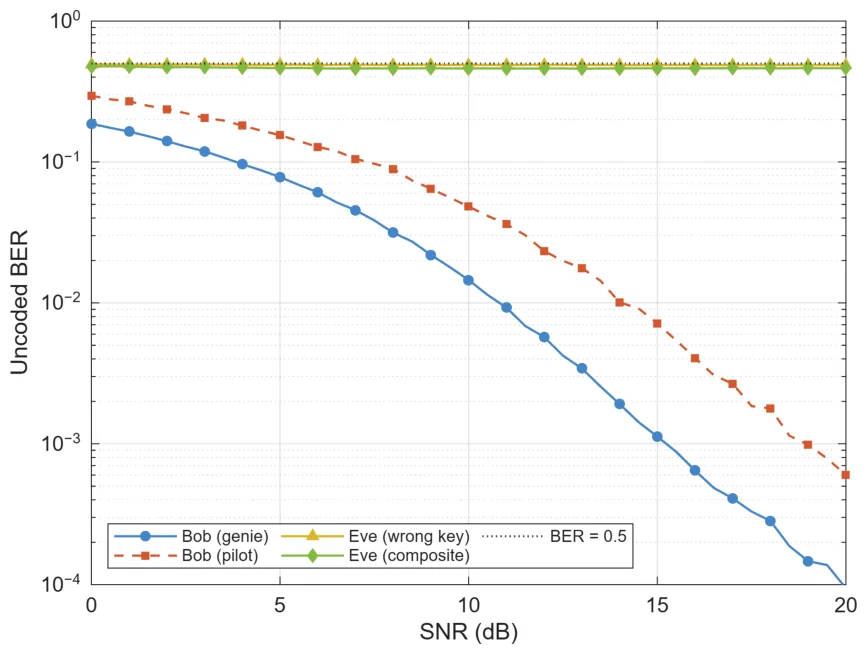
Uncoded BER vs. SNR (N=64, QPSK). Bob’s genie- and pilot-aided links fall steadily with SNR (below $2\times 10^{-4}$ and $6\times 10^{-4}$ respectively at 20 dB), while both eavesdropper receivers stay pinned near the 0.5 guess line (dotted). The wrong-key curve is flat in SNR, consistent with key-based obfuscation.

Figure 3 presents the BER results. Bob’s genie-aided BER drops rapidly with SNR, falling below $2\times 10^{-4}$ by 20 dB over n=248,000 bits (2000 trials/point). Bob’s pilot-estimated BER (using N=64 secret-pilot training symbols per channel estimate) shows a modest penalty of approximately 2–3 dB compared with the genie-aided case at low SNR, reaching $∼5\times 10^{-4}$ by 20 dB. Eve’s wrong-key BER stays within ±0.02 of 0.5 across all SNRs (range 0.483–0.492), a small residual negative bias at the edge of the finite-sample resolution. Any consistent bias away from 0.5 is exploitable structure (Section 4.1), not extra margin. Its magnitude is small enough that the naive receiver does not exploit it. The composite-channel Eve (who attempts to estimate G from the encrypted pilots without decryption) achieves BER between 0.46 and 0.48, biased slightly below 0.5 under this naive receiver due to the secret pilot phase interference described in Section 4.3. A sub-0.5 bias is not additional security: it indicates residual exploitable structure (Section 4.1), and the stronger-adversary experiment shows that a single known-plaintext frame suffices to collapse it.

Figure 3 shows two relevant trends. First, the wrong-key Eve curve is flat in SNR—her BER does not improve as her channel improves, unlike channel-advantage schemes whose security erodes when Eve’s SNR rises; this behavior stems from the unknown mask rather than a noise advantage and lets the threat model grant Eve a higher SNR than Bob without weakening the result. Second, the genie-to-pilot gap quantifies the cost of the secret-pilot estimator: at 0 dB the pilot noise inflates BER from 0.187 to 0.295, but the gap closes by 15 dB, and both fall below $10^{-3}$ by 18–20 dB—a low-SNR phenomenon, the regime where an outer code would operate anyway.

### 5.4. Legitimate-Link BER Benchmark

**Figure 4:**
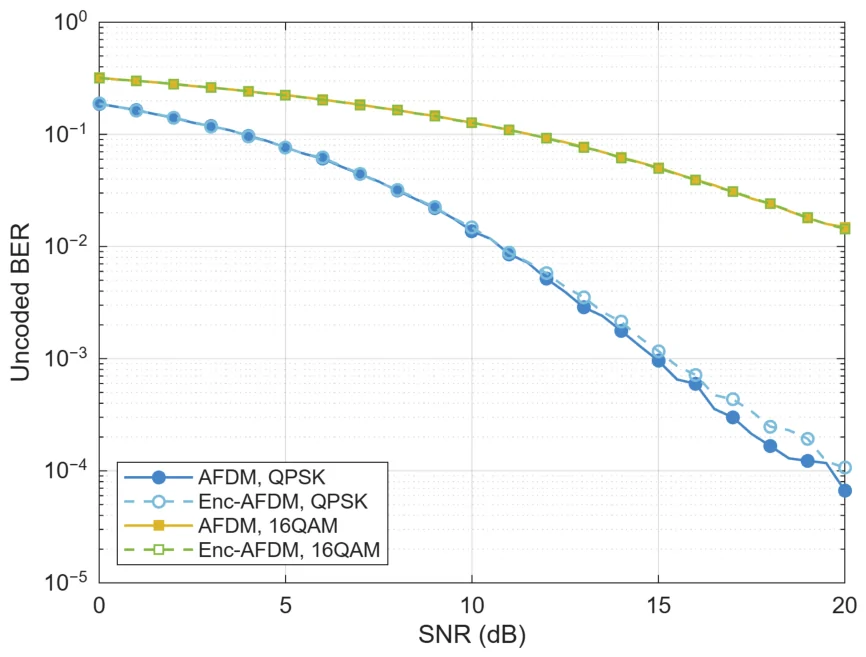
Legitimate-link BER for standard AFDM vs. Encrypted-AFDM (N=64, numerically known effective channel). The encrypted and unencrypted curves are visually indistinguishable for both QPSK and 16QAM (penalty <0.5 dB).

Figure 4 compares the BER performance of standard AFDM and Encrypted-AFDM for both QPSK and 16QAM. Across all SNRs and both modulations, the two BER curves overlap within Monte Carlo variation (Figure 4). Reading the residual horizontal gap between the curves at a fixed BER, the Encrypted-AFDM penalty is below 0.5 dB. These legitimate-link comparisons use a numerically known effective channel (denoted $H_{\mathrm{eff}}$ for standard AFDM and ${\tilde{H}}_{\mathrm{eff}}$ for Enc-AFDM, obtained by the column-probing procedure of Algorithm 1) for both waveforms. Pilot-estimated BER is characterized separately in the eavesdropper BER results above (QPSK, N=64). At several SNR points, Encrypted-AFDM slightly outperforms standard AFDM by an amount within Monte Carlo variation. The results confirm that time-domain encryption introduces negligible degradation to the legitimate link. This comparison is at equal raw SNR and does not account for Enc-AFDM’s pilot-training overhead. On a net-throughput basis, the two waveforms are not directly comparable. The spectral-efficiency cost of the *N*-block training cycle (Section 3.7) should be considered when interpreting the penalty claim.

### 5.5. Scalability to N = 128 with Fractional Doppler

**Figure 5:**
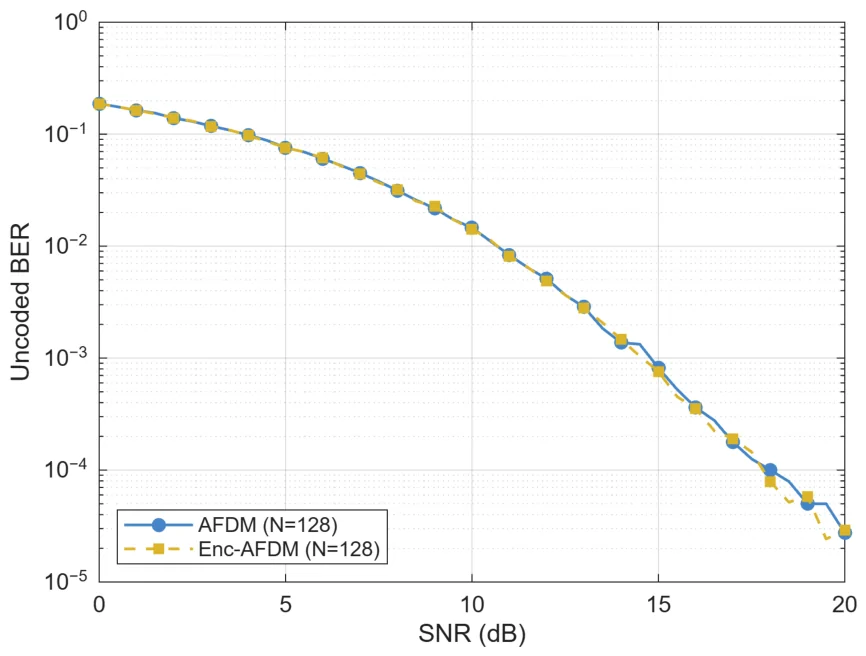
Legitimate-link BER for standard AFDM vs. Encrypted-AFDM (N=128, fractional Doppler ξ=0.3, QPSK, numerically known effective channel). The penalty remains below 0.5 dB.

Figure 5 shows that Encrypted-AFDM maintains a sub-0.5 dB penalty at N=128 with fractional Doppler (ξ=0.3, representing a maximum fractional component of 30% of the subcarrier spacing). The SNR penalty remains below 0.5 dB (numerically known effective channel). The key space at N=128 expands to $128!\times 2^{1024}$, representing a large mask parameterization space (the actual brute-force boundary is the 256-bit master-key entropy driving HMAC-SM3).

This block is the most demanding legitimate-link test. Fractional Doppler destroys the one-nonzero-per-row sparsity of $H_{\mathrm{eff}}$ (even before encryption), forcing the equalizer to invert a genuinely dense channel, and N=128 quadruples the $O(N^{2})$ estimation and $O(N^{3})$ equalization cost. That the penalty stays below 0.5 dB indicates the decrypt-first architecture and numerical $H_{\mathrm{eff}}$ construction do not break down at N=128. We restate the scope caveat. These figures use a numerically known effective channel, and pilot-estimated evidence remains limited to QPSK at N=64; the fractional-Doppler pilot-estimation case is not characterized here, and the larger key space is a brute-force statement only.

### 5.6. Encrypted Pilot Recovery NMSE

**Figure 6:**
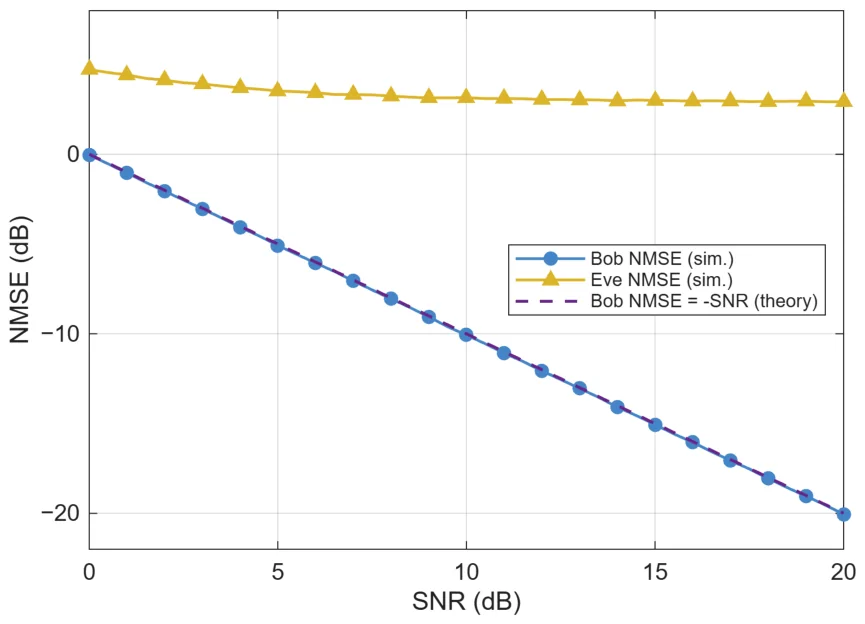
DAFT-domain symbol recovery NMSE. Bob’s NMSE tracks $-{\mathrm{SNR}}_{\mathrm{dB}}$ on a 1:1 slope, certifying model–simulator consistency; Eve’s stays flat near 3 dB (linear NMSE ≈2), the value for a prediction uncorrelated with an equal-power observation.

Figure 6 reports the NMSE between the received DAFT-domain pilot symbols and the model prediction. For Bob, the NMSE drops almost exactly with SNR (${\mathrm{NMSE}}_{\mathrm{Bob}}\approx -{\mathrm{SNR}}_{\mathrm{dB}}$), confirming that the model $y\approx H_{\mathrm{eff}}\;D\left({\mathrm{Enc}}_{K}\left(D^{-1}\left(x\right)\right)\right)$ accurately captures the encrypted transmission. For Eve, the NMSE remains constant at approximately 3.0 dB (NMSE ≈2.0 in linear scale), indicating that her wrong-key prediction captures roughly twice the signal power as error, an expected outcome when the model does not match the received signal.

The two NMSE curves are diagnostic rather than security metrics, and it is instructive to be precise about what each shows. Bob’s 1:1 NMSE-to-SNR slope certifies that the numerical effective-channel construction (Algorithm 1) is consistent with the time-domain channel simulator. Any modeling error would appear as an NMSE floor that fails to track SNR, and none is observed down to −30 dB. This is the concrete resolution of the analytical channel model mismatch. Eve’s flat ≈3 dB NMSE has a clean interpretation. A linear-scale NMSE of 2 is exactly what results when the prediction is uncorrelated with the observation of equal power, since $E∥y-\hat{y}{∥}^{2}={E∥y∥}^{2}+E∥\hat{y}{∥}^{2}=2\;E{∥y∥}^{2}$ for zero-mean uncorrelated equal-power vectors. Eve’s wrong-key model therefore carries essentially no information about her observation, consistent with the symbol decorrelation result below and the eavesdropper BER. NMSE, like BER, is a linear/second-order indicator and does not exclude higher-order structure exploitable by a stronger adversary.

### 5.7. Encryption Stage Ablation

Table 8 shows the ablation study using low-entropy keys (4 possible permutations × 4 QPSK-like phase values). Under “None” (no encryption), Eve’s BER matches Bob’s. With Scramble-only, Phase-only, or Both, Eve’s BER stays at approximately 0.5, while Bob’s BER drops to $∼10^{-4}$ at 20 dB. This indicates that for the tested low-entropy scenario, a single encryption stage (either scrambling or phase rotation) is sufficient to prevent wrong-key decoding. Two-stage encryption removes distinct residual structures: the permutation alone leaves per-sample phases intact (vulnerable to phase-based statistics), while the phase mask alone leaves sample ordering intact (vulnerable to ordering-based statistics). Retaining both stages eliminates both classes of residual structure simultaneously.

The deliberately low-entropy setting is what makes this ablation meaningful. With only four permutations and four phase values, a wrong key differs from the correct one in a small, enumerable way, so if a single stage were fragile it would show here. It does not; each stage alone already randomizes the wrong-key output, but we caution against over-reading this as “one stage suffices in general.” The two stages defend against different attack structures. The phase mask alone leaves the sample ordering intact (and hence any ordering-based statistic), while the permutation alone leaves per-sample phases intact (and hence any phase-based statistic). Retaining both is what removes both classes of residual structure simultaneously, which is why we keep the two-stage design despite the single-stage low-entropy result.

### 5.8. PAPR and Symbol Decorrelation

**Figure 7:**
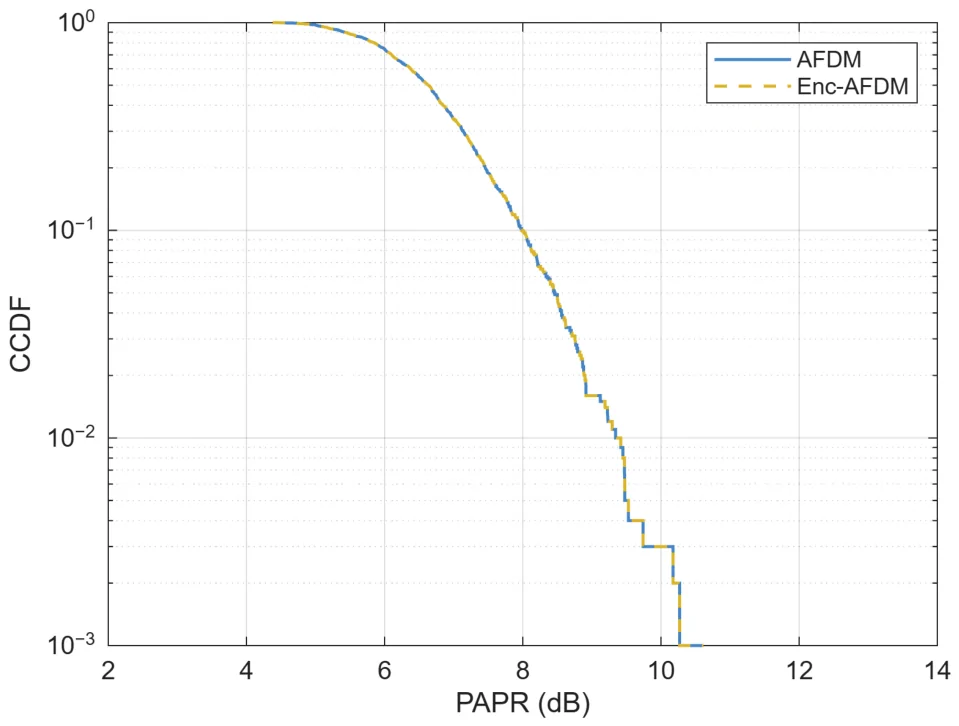
PAPR CCDF (N=64, QPSK, 1000 symbols). The AFDM and Enc-AFDM curves overlap exactly because the monomial mask preserves the multiset of sample magnitudes.

**Figure 8:**
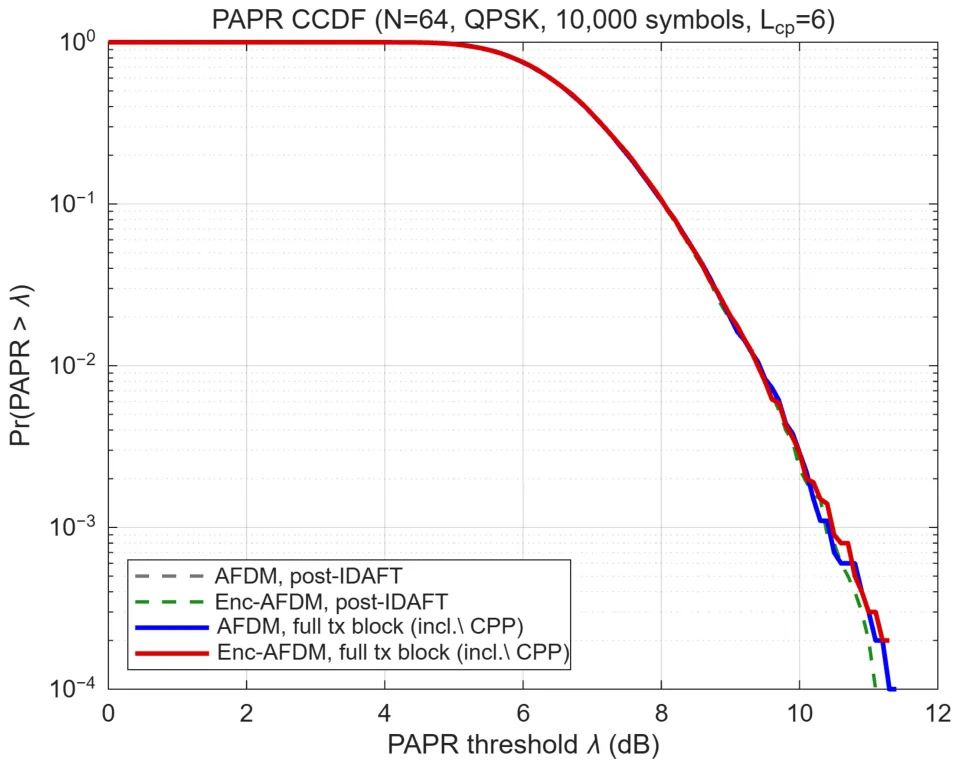
Transmit-block PAPR CCDF over the full $N+L_{\mathrm{cp}}$ samples that include the CPP, for N=64, $L_{\mathrm{cp}}=6$, QPSK, and 10,000 symbols. The Enc-AFDM and standard-AFDM curves agree closely in the 10,000-block Monte Carlo sample, with a mean of about 6.72 dB and a 99th percentile of about 9.3 dB, and both track the post-IDAFT CCDF. For the tested fixed key and parameters, no encryption-induced discrete-time, block-normalized PAPR penalty is observed after CPP inclusion.

The PAPR is preserved exactly: both AFDM and Encrypted-AFDM exhibit a mean PAPR of 6.68 dB (standard deviation 0.99 dB, 99th percentile 9.38 dB by the empirical percentile of the sample distribution) over 1000 QPSK symbols, as shown in Figure 7. This follows from (15): the monomial mask (permutation plus per-sample phase rotation) preserves the multiset of sample magnitudes exactly. The practical consequence is that the encryption adds no post-IDAFT PAPR penalty. It does not increase the PAPR of the *N* post-IDAFT samples and so shows no additional PAPR penalty on those samples, and any PAPR-reduction technique validated for AFDM is preserved because the mask only reorders and phase-rotates an already-fixed magnitude multiset. To address the full transmit block directly, Figure 8 reports the PAPR CCDF over the $N+L_{\mathrm{cp}}$ samples that include the CPP. The Enc-AFDM and standard-AFDM full-block curves agree closely in the 10,000-block Monte Carlo sample. Both have a mean transmit-block PAPR of about 6.72 dB and a 99th percentile of about 9.3 dB, consistent with the 6.68 dB post-IDAFT value of Figure 7, and both track the post-IDAFT CCDF. The observed closeness is consistent with the small prefix ratio $L_{\mathrm{cp}}/N=6/64$, and no additional discrete-time, block-normalized PAPR penalty is observed under the tested configuration. Key-to-key variation is not characterized by this single-key experiment. Establishing full high-power amplifier (HPA) operating-point invariance would further require oversampled CCDFs and error-vector-magnitude and adjacent-channel-leakage-ratio measurements beyond the present discrete-time evaluation.

**Figure 9:**
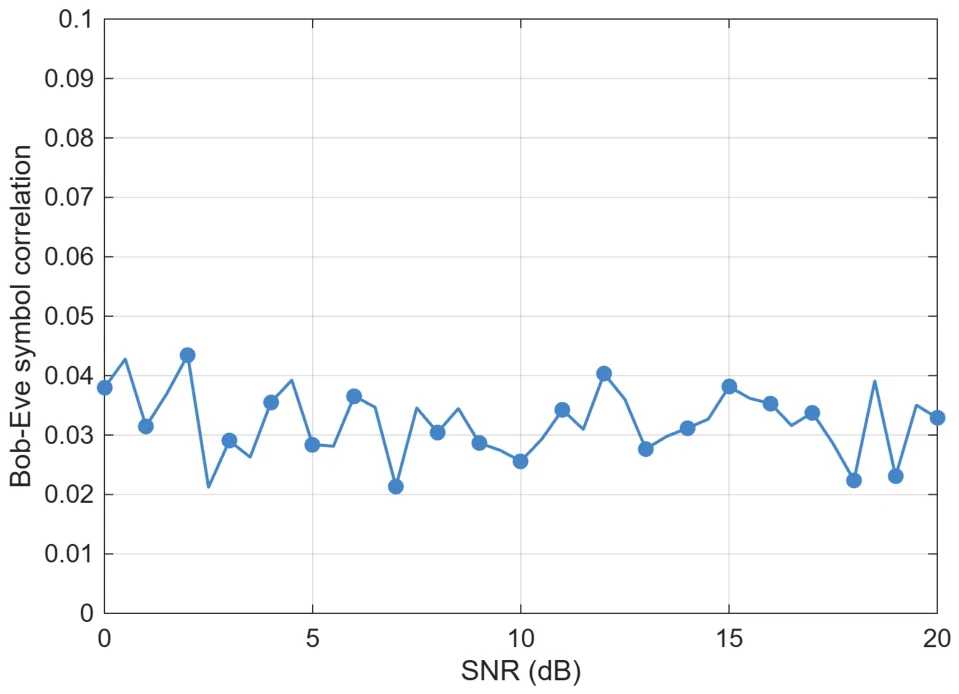
Bob–Eve decoded symbol Pearson correlation vs. SNR (N=64, QPSK). The correlation remains below 0.044 at all SNRs, confirming that Eve’s soft symbol estimates carry no linear information about Bob’s.

The Bob–Eve decoded symbol Pearson correlation (real part) remains below 0.044 at all SNRs (range: 0.021 to 0.044), as shown in Figure 9, confirming that the eavesdropper’s symbol estimates carry no detectable linear correlation with the legitimate receiver’s. The symbol-correlation experiment provides an independent confirmation of the wrong-key obfuscation: not only is Eve’s BER at chance, but her soft symbol estimates carry no linear information about Bob’s, consistent with the structurally random-unitary argument of Section 4. Vanishing linear correlation does not preclude nonlinear dependence exploitable by a more sophisticated estimator. This condition is necessary but not sufficient for full confidentiality.

The NMSE, ablation, PAPR, and correlation experiments support the same interpretation under the tested receivers, each of which would independently fail if the encryption were either ineffective against Eve or destructive to Bob. Their agreement is why we consider the legitimate-link/attacker-link separation consistent across the passive adversaries evaluated here, even as we decline to extend it to the coded or decision-directed adversaries of Section 6.

### 5.9. Multi-Frame Known-Plaintext Attack

**Figure 10:**
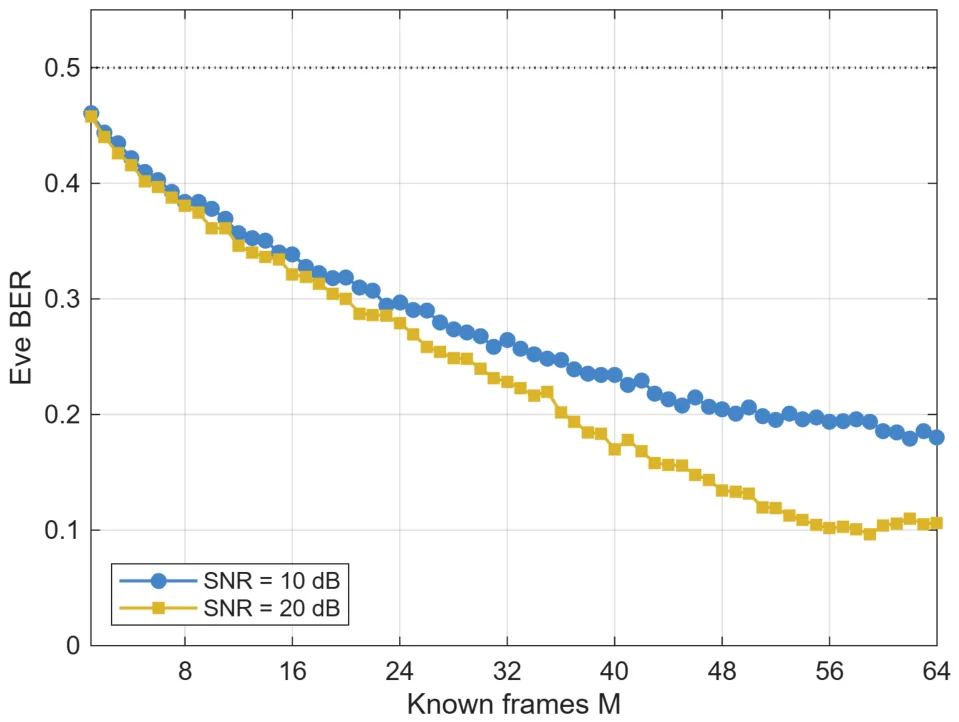
Eve’s BER under the multi-frame known-plaintext LS attack vs. the number of fully known frames *M* (M=1,2,…,N, N=64). Leakage grows steadily away from the 0.5 guess line (dotted) well before M=N: there is no safe M≪N plateau. This characterizes the naive A3 adversary; the operative security requirement is set by the stronger A4 adversary of the stronger-adversary experiment (per-frame nonce-derived pilots, Section 4.5).

Figure 10 reports the known-plaintext LS attack result. As analyzed in Section 4.4, under the LS-based composite channel recovery attack (24), Eve’s BER decreases with the number of known frames *M*. With one known frame (M=1), Eve’s BER is 0.458 at 20 dB (barely better than random guessing). With M=16, it drops to 0.321. At M=N=64, Eve’s BER falls to 0.180 at 10 dB and 0.106 at 20 dB—a severe degradation, though not driven to zero over the finite trial budget. Protection is not binary in *M*. Leakage grows steadily (BER 0.458→0.416→0.321 for M=1,4,16 at 20 dB), so even M≪N produces an exploitable bias. The practical implication is that the key must be refreshed well before *N* fully known frames can accumulate; the admissible refresh interval is dictated by the maximum tolerable leakage, not by the M=N break point. Following the methodology above, these BER values carry two-sided 95% Wilson intervals at the block’s *M*-dependent budget; the intervals widen as *M* increases and the per-point frame budget shrinks, reaching about [0.090,0.124] at *M* = *N* = 64 and 20 dB ($\hat{p}=0.106$). The leakage trend—BER decreasing steadily with *M* and falling well below 0.5 by *M* = *N*—is robust to this interval width, which is why the trend rather than the exact BER value is the operative takeaway.

### 5.10. Key-Reuse Attack

**Figure 11:**
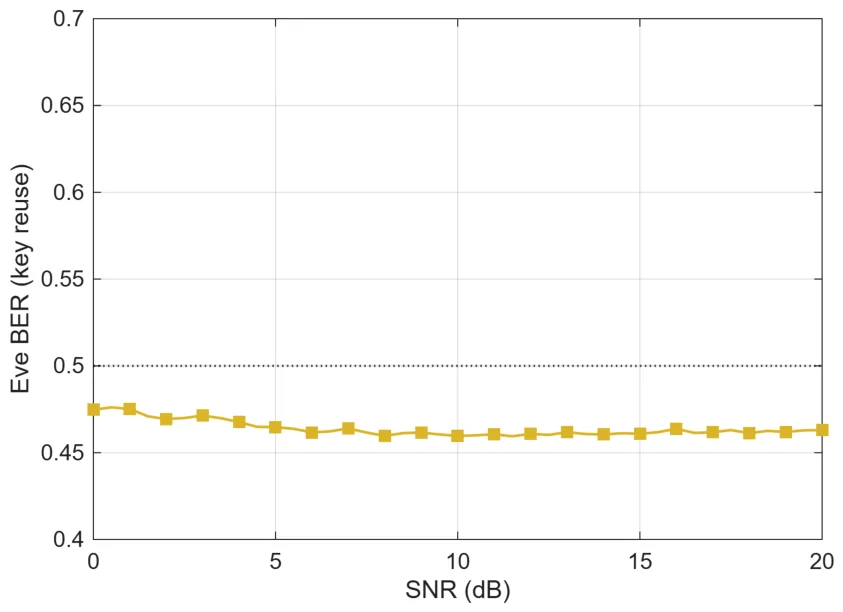
Key-reuse attack (N=64, QPSK). Same key and channel across two frames; Eve estimates the composite channel from Frame 1 and applies the estimate to Frame 2. BER stays near (just below) 0.5 at all SNRs; naive reuse remains phase-rotated and fails to decode.

With the same key and identical channel across two frames, Eve’s BER values on Frame 2 using Frame 1’s composite channel estimate are 0.475, 0.465, 0.460, 0.461, and 0.463 at SNRs 0, 5, 10, 15, 20 dB, respectively (Figure 11) (two-sided 95% Wilson interval at the block’s ∼50-frame budget, e.g., [0.449,0.477] at 20 dB, so the upper bound sits just below 0.5). The BER stays just below 0.5 at all SNRs; the naive reuse of a secret-pilot composite estimate across frames remains phase-rotated and fails to decode (a sub-0.5 bias is residual exploitable structure, not extra security). This does not establish resistance to joint multi-frame key-reuse attacks. The nonce requirement from the stronger-adversary experiment still applies. Per-frame nonce-derived pilot phases are the specified mitigation; they are validated against the cross-frame anchor in Section 5.12, while key-reuse and joint multi-frame attacks remain unquantified.

### 5.11. Stronger Passive Adversary: Blind Phase Recovery

**Figure 12:**
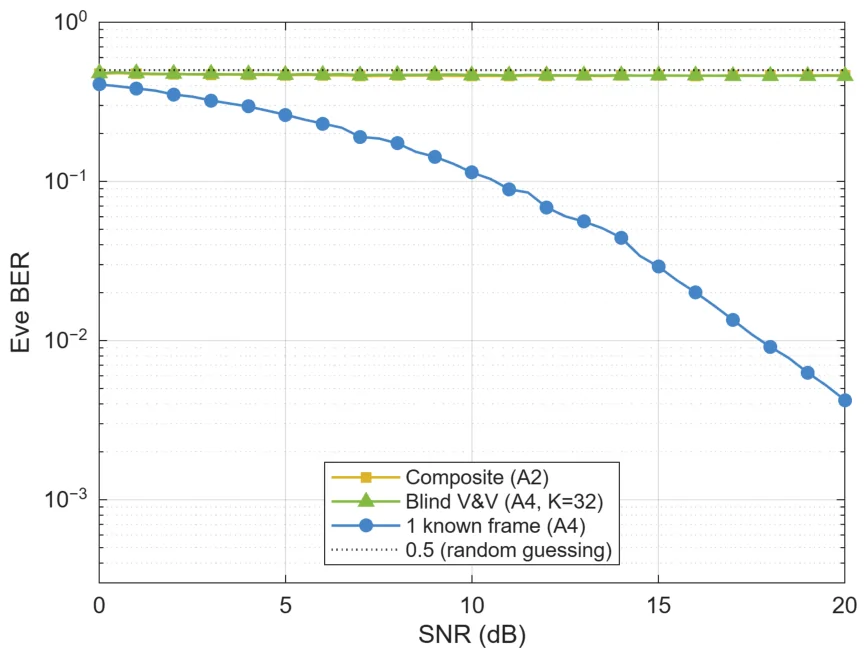
Stronger passive adversary (N=64, QPSK). Blind Viterbi–Viterbi phase recovery over K=32 frames stays at the composite level near the 0.5 line (dotted); the QPSK π/2 ambiguity is unresolvable blind, whereas a single known-plaintext frame collapses Eve’s BER with SNR. Hence, per-frame nonce-derived pilots are mandatory. The dotted horizontal line marks random guessing (BER =0.5).

Figure 12 evaluates the stronger passive adversary of class A4 (Section 4.5), which combines the composite estimate GΘ with blind phase recovery. The composite (A2) column is recomputed here under its own trial budget (*n* = 4960 bits/point) and random seed, which accounts for its slight numerical difference from the composite-Eve curve in Figure 3; both lie in the same 0.46–0.48 range (a small bias below 0.5) and carry the same interpretation. Two results stand out. First, a purely blind adversary applying per-subcarrier fourth-power (Viterbi–Viterbi) phase estimation over K=32 observed same-key frames fails: her BER stays at 0.46–0.48, statistically indistinguishable from the naive composite receiver. The four-fold rotational symmetry of QPSK leaves a residual π/2 ambiguity that blind processing cannot break. Second, a single known-plaintext frame resolves the ambiguity completely: Eve’s BER then falls from 0.41 at 0 dB to $1.1\times 10^{-1}$ at 10 dB and $4.2\times 10^{-3}$ at 20 dB (two-sided 95% Wilson interval at the ∼4960-bit budget, e.g., [0.0027,0.0064] at 20 dB). This single-frame boundary is far tighter than the naive *N*-frame LS boundary of the multi-frame KPA experiment above, because the pilot-derived composite estimate reduces the attack to resolving *N* residual phases. It is the operative security requirement: per-frame nonce-derived pilots are mandatory, since otherwise one known preamble frame suffices to break composite-channel confidentiality (Section 4.5).

### 5.12. Nonce-Defense Validation

**Figure 13:**
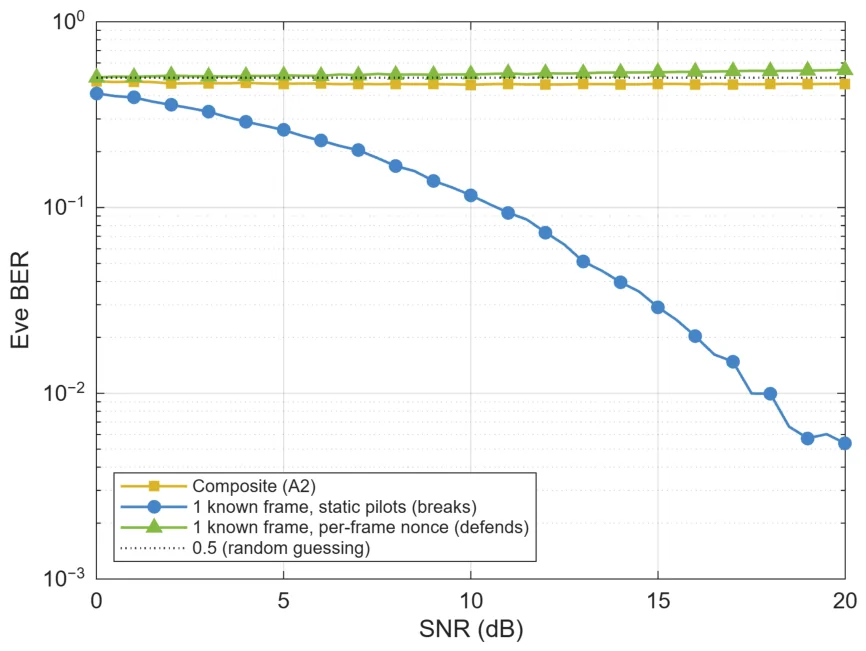
Per-frame nonce pilots defeat the cross-frame anchor attack (N=64, QPSK). With static pilots, one known frame resolves the per-subcarrier phase and Eve’s BER on an unknown data frame collapses with SNR (blue); with per-frame nonce-derived pilots, the resolved phase does not transfer across frames, and the BER stays at ≈0.5 (green), indistinguishable from the naive composite receiver (yellow). The dotted horizontal line marks random guessing (BER =0.5).

To complete the stronger-adversary evaluation, Figure 13 validates the per-frame-nonce defense against the single-known-frame (A4) anchor attack of Section 4.5. Eve resolves the per-subcarrier phase from one fully known anchor frame and applies it to de-rotate an unknown data frame. With static pilots (the setting of Figure 12), the resolved phase transfers across frames and Eve’s BER collapses with SNR, to $5.4\times 10^{-3}$ at 20 dB. With per-frame nonce-derived pilots, the phase resolved at the anchor’s nonce $t_{a}$ does not match the data frame’s nonce $t\neq t_{a}$, the de-rotation fails, and Eve’s BER stays in the near-random regime across all SNRs (0.50–0.55; the two-sided 95% Wilson interval at the ∼40-frame budget is, e.g., [0.505,0.535] at 20 dB, whose lower bound sits just above 0.5), a small residual above-chance bias rather than extra security per Section 4.1. The cross-frame anchor attack is thus defeated. It does not protect a frame that itself contains enough known plaintext (e.g., standardized headers or cyclic redundancy check (CRC) fields) to resolve the per-subcarrier phase within that frame—this same-frame boundary is the key open question (Section 4.5).

### 5.13. Delay–Doppler Sensing Preservation

**Figure 14:**
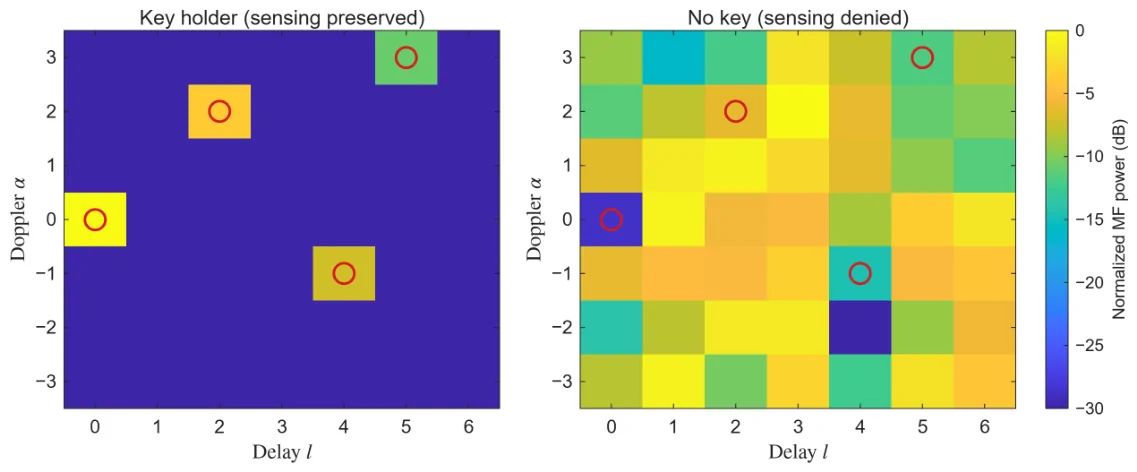
Delay–Doppler matched-filter power maps (N=64, four scatterers, noiseless). (**Left**) key holder recovers the standard AFDM effective channel; all matched-filter energy concentrates at the true target cells (sensing concentration =0 dB, 2/4 targets above −6 dB threshold). (**Right**) no-key receiver observes the composite channel $G=H_{\mathrm{eff}}P$; energy is spread uniformly (sensing concentration =−16.6 dB, 0/4 targets detected). Red circles mark true scatterer locations. The color scale (colorbar) shows the normalized matched-filter power in dB.

Figure 14 visualizes the delay–Doppler matched-filter maps for the key holder and a no-key receiver (Section 4.6). Red circles mark the four true scatterer locations. The key holder’s map shows sharp peaks at the true target cells; the no-key receiver’s map is a uniform noise floor with no detectable peaks. This confirms that the unitary time-domain mask preserves AFDM’s ISAC sensing capability for the authorized receiver while denying it to an eavesdropper.

**Figure 15:**
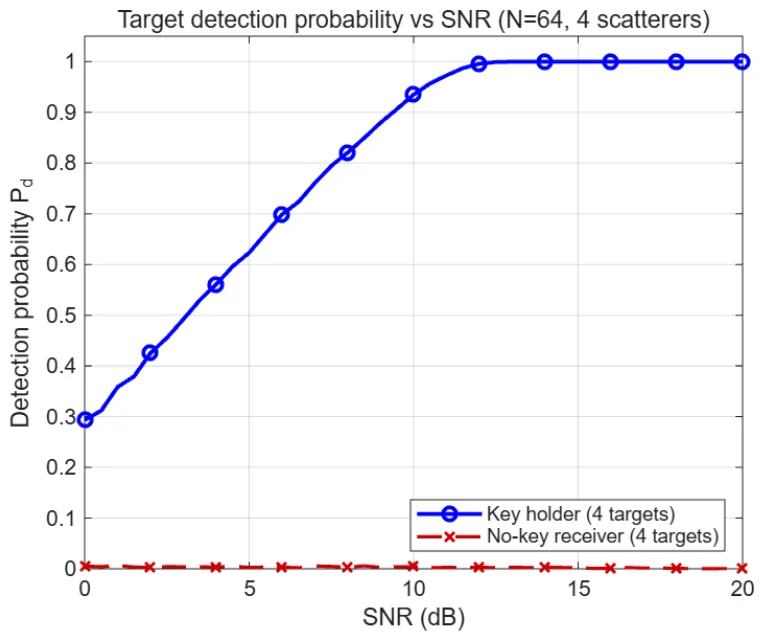
Target detection probability vs. SNR (N=64, four scatterers, non-coherent energy detector with threshold at mean +3σ of non-target cells). The key holder’s detection probability rises with SNR and reaches 1.0 by 18 dB. The no-key receiver’s target-cell exceedance rate stays at approximately 0.005 across all SNRs.

Figure 15 extends the sensing evaluation to noisy conditions. For each candidate delay–Doppler pair (l,α) the receiver computes the total power along the corresponding shifted diagonal of the estimated effective channel, $\sum_{p}{\left|{\hat{H}}_{\mathrm{eff}}\left[p,\left(p+\mathrm{loc}\right)\;\mathrm{mod}\;N\right]\right|}^{2}$, where $\mathrm{loc}=(-\alpha +2Nc_{1}l)\;\mathrm{mod}\;N$. A threshold at the mean plus three standard deviations of the non-target cells is then applied to each of the four true target locations in this fixed-scene evaluation. The key holder’s detection probability rises from 0.29 at 0 dB to 0.91 at 10 dB and saturates at 1.0 by 18 dB. The no-key receiver’s target-cell exceedance rate stays at approximately 0.005 across all SNRs. The time-domain mask therefore preserves sensing for the authorized receiver under practical noise in this configuration.

**Figure 16:**
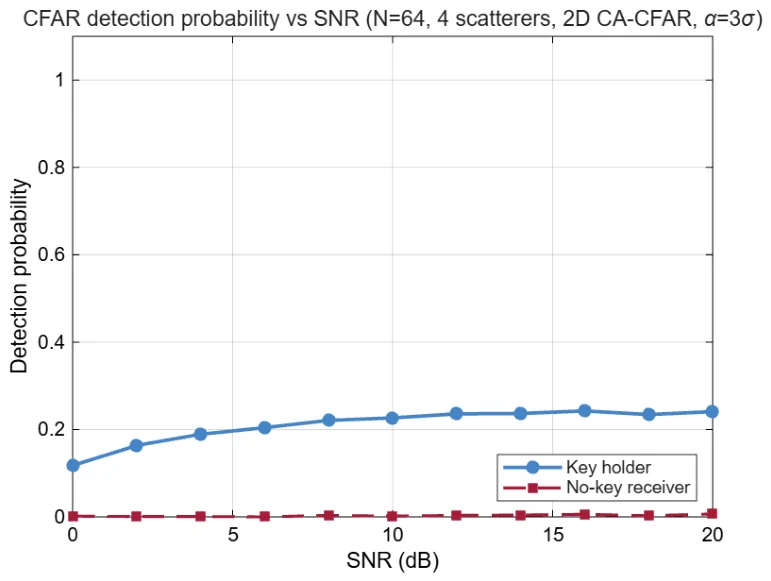
CFAR detection probability vs. SNR (N=64, four scatterers, 2D CA-CFAR with 3σ threshold). The key holder’s $P_{d}$ rises with SNR; the no-key receiver’s stays at approximately 0.02. The CFAR replaces the oracle-aided threshold of Figure 15 with an operational detector that does not assume knowledge of target locations.

To address the concern that the above threshold uses knowledge of the true target locations, we also evaluated an operational 2D cell-averaging CFAR detector with a (±1,±1) guard window and a 3σ threshold (Figure 16). The key holder’s CFAR detection probability rises from 0.12 at 0 dB to 0.24 at 20 dB, while the no-key receiver stays at approximately 0.02 across all SNRs. The CFAR $P_{d}$ is lower than the oracle-aided value because the adaptive threshold is more conservative, but the qualitative separation is preserved ($P_{\mathrm{fa}}<0.01$ for the no-key receiver).

**Figure 17:**
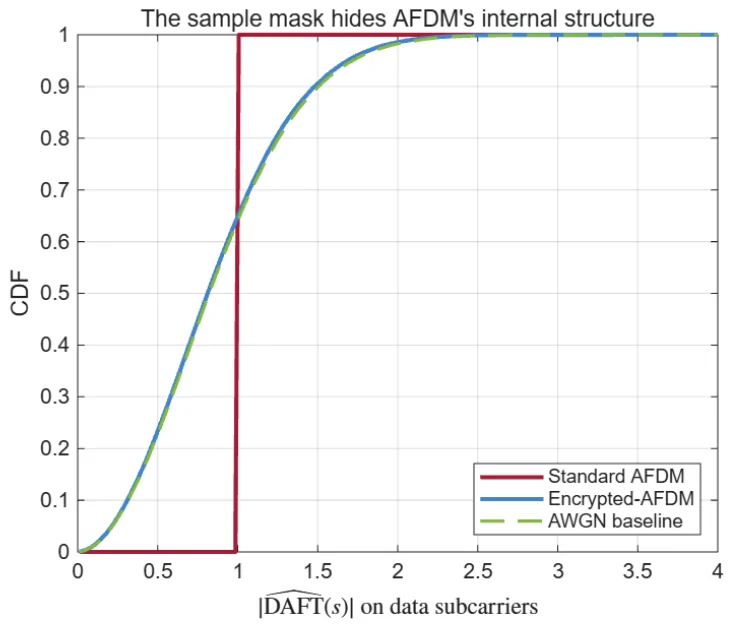
The sample mask hides AFDM’s internal structure. CDF of $\left|\hat{\mathrm{DAFT}}\left(s\right)\right|$ on data subcarriers over 2000 symbols. Standard AFDM is concentrated at the QPSK radius $1/\sqrt{2}$ (CoV=0); Encrypted-AFDM is spread and matches the AWGN baseline (CoV≈0.52). Under this DAFT-magnitude statistic, the encrypted waveform is indistinguishable from AWGN.

We test whether the time-domain mask hides AFDM’s recognizable sample-level structure. Figure 17 measures this directly: we apply AFDM-aware processing (the DAFT) to the time-domain signal and examine $\left|\hat{\mathrm{DAFT}}\left(s\right)\right|$ on the data subcarriers. For standard AFDM, the DAFT recovers the QPSK constellation, so $\left|\hat{\mathrm{DAFT}}\left(s\right)\right|$ is constant (coefficient of variation CoV=0). After the key-derived sample mask, the DAFT output is spread: its magnitude distribution matches AWGN (CoV≈0.52 for both, versus 0 for AFDM), i.e., under this DAFT-magnitude statistic the encrypted waveform is indistinguishable from AWGN. The mask therefore suppresses the internal structure a waveform recognizer would exploit; a full modulation-classifier evaluation is left to future work.

### 5.14. Comparison with Parameter-Domain Encryption

We benchmark Encrypted-AFDM against CP-AFDM, a parameter-domain scheme that permutes the DAFT-domain subcarriers from a shared key [14]. The comparison uses the same channel model, QPSK modulation, N=64, integer Doppler, and a numerically known effective channel for both schemes.

**Figure 18:**
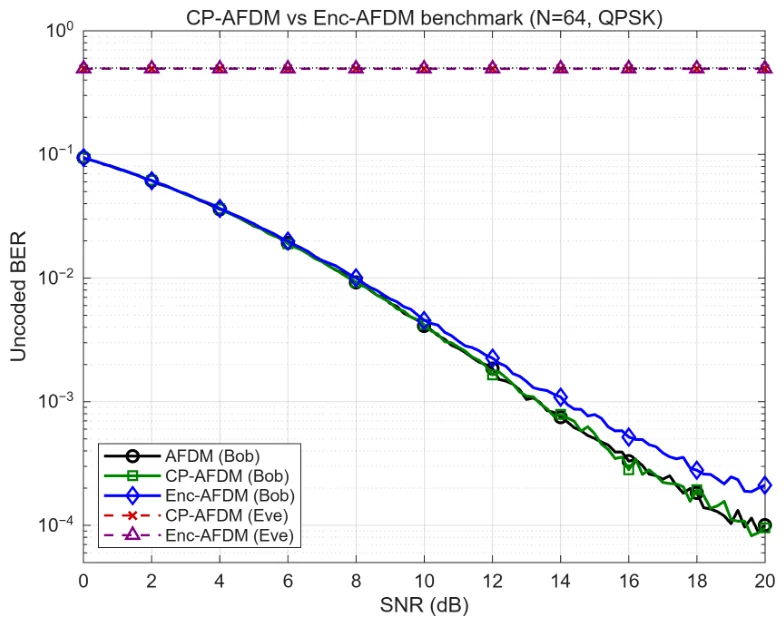
BER comparison of standard AFDM, CP-AFDM, and Encrypted-AFDM (N=64, QPSK, integer Doppler, numerically known effective channel). The three legitimate-link curves overlap within Monte Carlo variation. Both eavesdropper curves stay near 0.5. Mean PAPR is approximately 6.7 dB for all three waveforms.

Figure 18 shows the uncoded BER. The legitimate-link curves of standard AFDM, CP-AFDM, and Encrypted-AFDM overlap within Monte Carlo variation at all tested SNRs, confirming that both encryption approaches introduce negligible BER penalty. The eavesdropper curves of both CP-AFDM and Encrypted-AFDM stay near 0.5 across all SNRs, confirming that both block a wrong-key receiver. The mean PAPR of all three waveforms is approximately 6.7 dB (within 0.02 dB), consistent with the PAPR-preservation analysis of Section 3.7.

The two schemes differ in mechanism and overhead rather than in BER or PAPR. CP-AFDM permutes in the DAFT domain and preserves the sparse effective channel, so the legitimate receiver can use standard sparse channel estimation. Encrypted-AFDM permutes in the time domain, which densifies the effective channel and requires the full-band estimator and dense equalization of Section 3.10. In return, Encrypted-AFDM applies the time-domain mask that makes the transmitted waveform approach the AWGN baseline under the evaluated DAFT-magnitude statistic under DAFT-magnitude inspection (Figure 17) and introduces the secret-pilot defense against composite-channel estimation (Section 4.3). Rou et al. do not include this defense in their scheme. The choice between the two schemes is therefore one of trading computational simplicity for waveform-level confidentiality.

**Figure 19:**
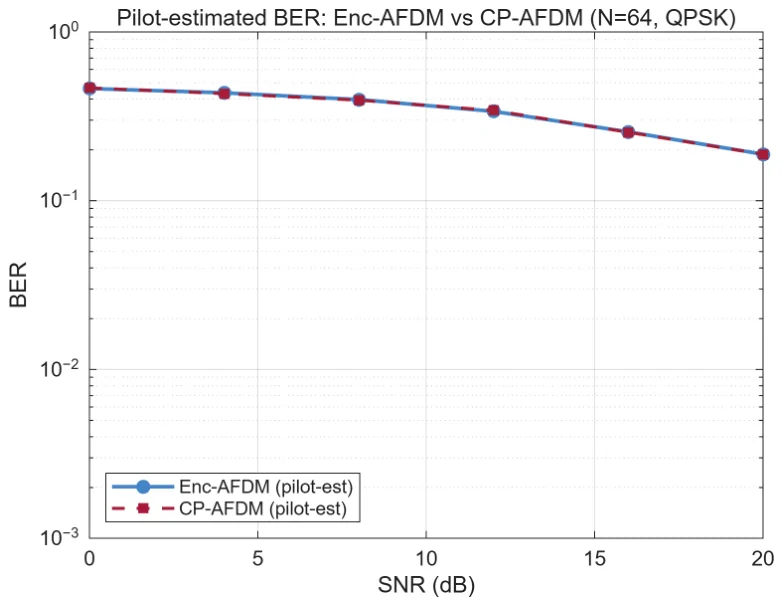
Pilot-estimated BER: Encrypted-AFDM vs. CP-AFDM (N=64, QPSK, same *N*-block noisy-pilot training). The two curves overlap, confirming no BER penalty from time-domain encryption when both schemes use equal training overhead.

To verify that the comparison is fair, we also evaluated both schemes under pilot-estimated channels rather than oracle CSI. Both use the same *N*-block full-band training with noisy pilots and LMMSE equalization, so the training overhead is identical. Figure 19 shows that the two schemes perform nearly identically under pilot estimation (BER within 0.01 across 0–20 dB), confirming that the encryption introduces no BER penalty relative to CP-AFDM when both face equal training overhead.

### 5.15. Timing Synchronization Sensitivity

**Figure 20:**
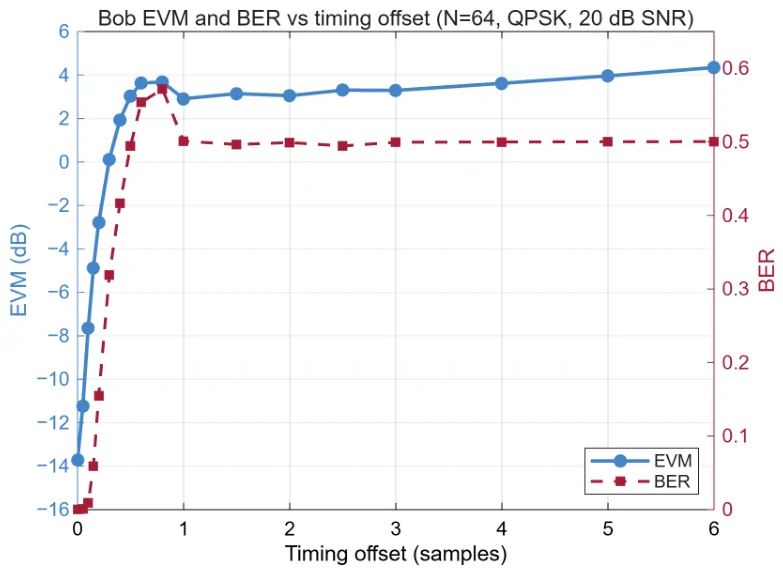
Bob EVM and BER vs. timing offset (N=64, QPSK, 20 dB SNR; offset modeled as a residual circular shift within the CPP guard). At 0 samples offset, EVM is −13.7 dB and BER is $2\times 10^{-4}$. The BER rises smoothly over 0–0.5 samples and saturates near 0.5 for offsets of half a sample or more, while the EVM rises from −13.7 dB to about 3–4 dB, because the time-domain permutation is misaligned.

The time-domain permutation R is position-dependent, so a timing offset at the receiver misaligns the permutation and can destroy the signal. Figure 20 quantifies this sensitivity, modeling the offset as a residual timing error within the CPP guard (which the CPP renders as a circular shift of the received block). At 20 dB SNR with no offset, Bob achieves an error vector magnitude (EVM) of −13.7 dB and BER of $2\times 10^{-4}$. The BER rises smoothly with the offset over 0–0.5 samples (approximately 0.009 at 0.1 samples, 0.16 at 0.2, and 0.49 at 0.5) and saturates near 0.5 for offsets of half a sample or more, while the EVM rises from −13.7 dB to about 3–4 dB. The EVM is mildly non-monotonic in this saturated regime—it peaks around a sub-sample offset, eases slightly near an integer offset, and drifts upward as the offset grows toward the CPP length—because once the BER is at chance the EVM no longer measures decodable symbol quality but the residual energy at the output of Bob’s zero-offset equalizer driven by the offset-perturbed signal: a sub-sample circular shift spreads the integer-position-indexed samples and decorrelates them most strongly, an integer shift is a cleaner (mis)permutation that retains a little more structure, and a larger offset accumulates more perturbation toward the guard edge. The operationally relevant metric is the BER, which stays at 0.5 throughout this regime, so the EVM fine-structure there does not affect the conclusion. The permutation is thus sensitive to sub-sample timing offsets, which the receiver must correct before decryption: half a sample or more effectively denies the link. The permutation makes the scheme slightly less tolerant of fractional offsets than standard AFDM because the mask is integer-position-indexed. In practice, the fractional-delay correction and frame synchronization already required by any AFDM receiver would be applied before decryption.

### 5.16. Pilot Jamming and Timing Sensitivity

**Figure 21:**
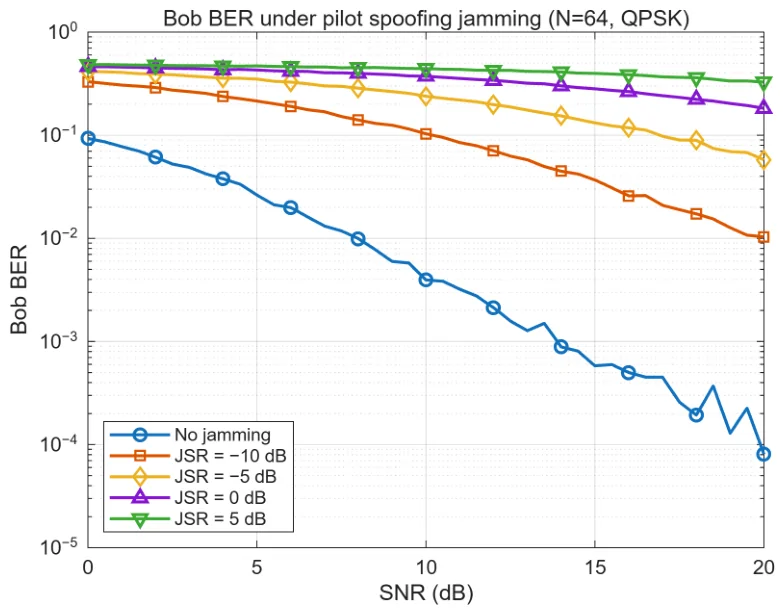
Bob BER under additive pilot jamming at various JSR levels (N=64, QPSK). At −10 dB JSR, the link remains functional. At 0 dB JSR, the pilot estimation is overwhelmed at low SNR. At +5 dB JSR, the link is effectively denied at low SNR.

An active adversary may inject jamming noise on Bob’s pilot observations to corrupt the channel estimate. This targets the training phase, not the encryption. Figure 21 shows Bob’s BER under additive pilot jamming at various jammer-to-signal ratios (JSR). At −10 dB JSR, Bob still achieves BER below 0.15 at 8 dB SNR. At 0 dB JSR, Bob’s BER degrades to approximately 0.4 at low SNR but improves to roughly 0.18 at 20 dB as the data SNR compensates for the pilot noise. At +5 dB JSR, the link is effectively denied at low SNR, though at 20 dB data SNR the BER drops to approximately 0.3. Pilot jamming is thus an availability threat rather than a confidentiality break. The mask itself is not compromised, but the channel estimate becomes unreliable. This experiment models additive Gaussian jamming, not coherent pilot spoofing (structured injection designed to bias the estimated channel coefficients), which would require a separate analysis. Mitigation through pilot authentication and jamming-resistant training is orthogonal to the time-domain mask. We also evaluated the eavesdropper’s BER under pilot interference at the same JSR levels (−10, 0, +5 dB). Eve’s BER stays at approximately 0.50 at all JSR values, confirming that pilot interference does not help Eve resolve the secret pilot phases or decode the data. The spoofing degrades Bob’s availability but does not improve Eve’s confidentiality attack.

### 5.17. Coded and Decision-Directed Adversary

**Figure 22:**
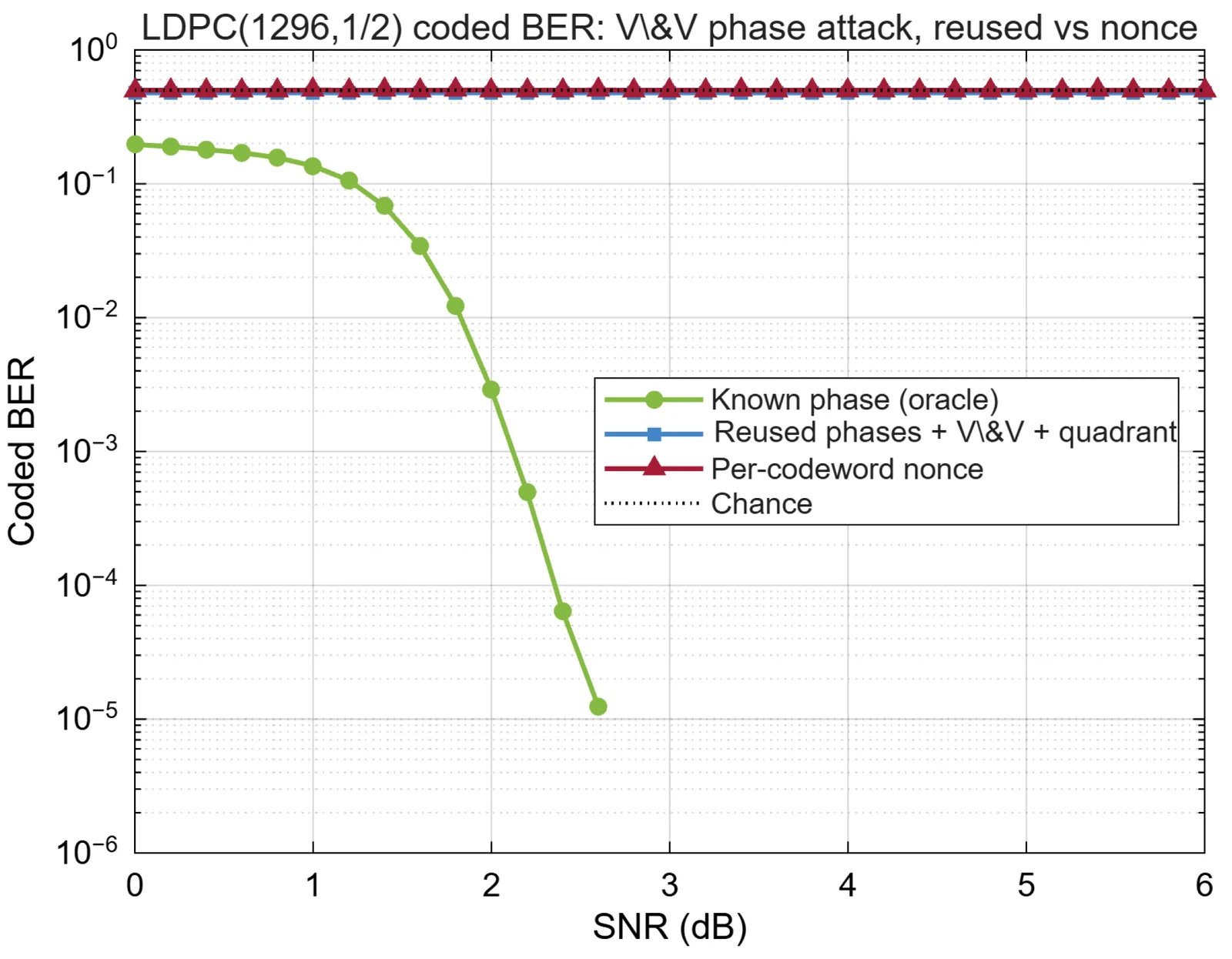
LDPC(1296,1/2) coded BER under three phase-estimation conditions ($10^{7}$ information bits for the oracle, $10^{6}$ for the attacks, 0.2 dB steps, 25 min-sum iterations). With known phase (oracle), the code resolves a clean waterfall to $1.2\;\times \;10^{-5}$ at 2.6 dB and to zero above 2.8 dB. Under reused pilot phases with V&V estimation (K=8 training codewords) and quadrant search, BER stays at ≈0.48 due to the QPSK π/2 rotational symmetry. Under per-frame nonces, BER stays at 0.50.

To test whether a strong outer code helps Eve resolve the per-subcarrier phase ambiguity, we evaluate a WiFi LDPC code (block length 1296, rate 1/2, min-sum decoding, 25 iterations) under three conditions (Figure 22). The oracle curve, which carries a real waterfall, is run with $10^{7}$ information bits per SNR point and the two attack conditions with $10^{6}$, both in 0.2 dB steps. With known pilot phases (oracle), the LDPC decoder resolves a clean waterfall: coded BER falls from 0.20 at 0 dB through $2.9\;\times \;10^{-3}$ at 2 dB to $1.2\;\times \;10^{-5}$ at 2.6 dB and reaches zero above 2.8 dB, confirming the code functions correctly. Under reused pilot phases with V&V fourth-power phase estimation trained on K=8 codewords plus a four-quadrant search, the coded BER stays at approximately 0.48—the π/2 rotational symmetry of QPSK prevents reliable phase resolution even with coding gain. Under per-frame nonces (one observation per phase), the coded BER stays at 0.50. The LDPC coding gain does not help Eve resolve the per-subcarrier phase ambiguity for constant-modulus signaling, confirming the analytical identifiability result of Section 4.5 with a concrete code.

**Figure 23:**
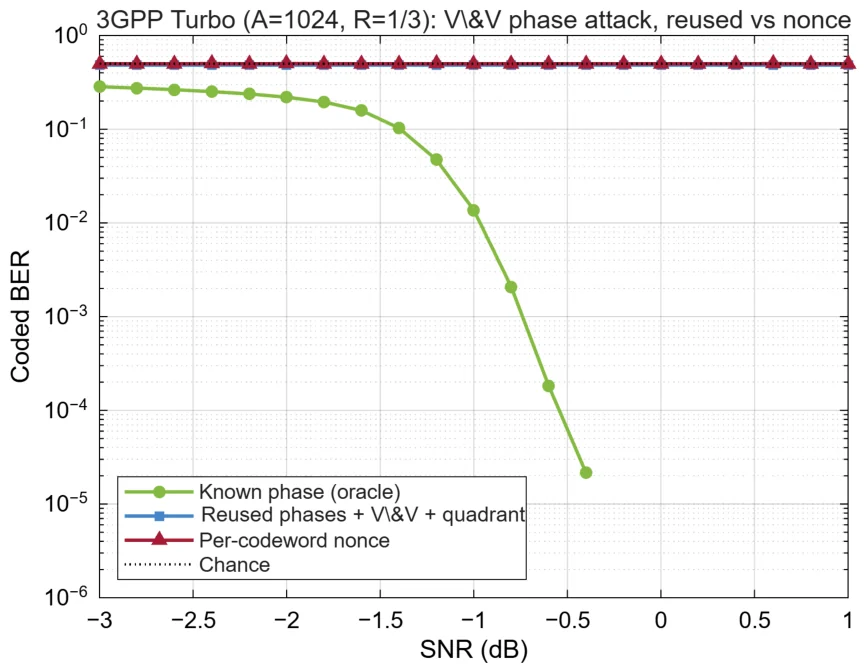
3GPP/LTE Turbo (A=1024, R=1/3) coded BER under three phase-estimation conditions ($10^{7}$ information bits for the oracle, $10^{6}$ for the attacks, 0.2 dB steps). With known phase (oracle), turbo resolves a sharp waterfall to $2.2\;\times \;10^{-5}$ at −0.4 dB and to zero above −0.2 dB. Under reused pilot phases with V&V and quadrant search, BER stays at ≈0.48; under nonces, at 0.50. The per-subcarrier phase ambiguity requires $4^{N}$ quadrant combinations, so neither turbo nor LDPC coding gain breaks it.

A 3GPP/LTE Turbo code (A=1024, rate 1/3 mother code, 8 iterations) gives a consistent result (Figure 23), again with $10^{7}$ information bits for the oracle and $10^{6}$ for the attacks in 0.2 dB steps. With known phase, the turbo decoder resolves a sharp waterfall: coded BER falls from 0.28 at −3 dB through $1.4\;\times \;10^{-2}$ at −1 dB to $2.2\;\times \;10^{-5}$ at −0.4 dB and reaches zero above −0.2 dB. Under reused pilot phases with V&V estimation and quadrant search, the coded BER stays at approximately 0.48; under per-frame nonces, at 0.50. The reason the coding gain cannot help is that the per-subcarrier phase ambiguity is not a single global rotation but *N* independent modulo-π/2 ambiguities; a global quadrant search can try only 4 rotations, while the correct assignment requires resolving $4^{N}$ combinations—computationally infeasible for any realistic *N*. Neither LDPC nor Turbo coding gain can break the per-subcarrier phase ambiguity for constant-modulus signaling.

## 6. Discussion

The simulation study of Section 5 establishes feasibility under a specified set of adversaries. This section discusses deployment requirements, composability with parameter-domain methods (Section 2), and the open problems that bound practical value. We separate experimentally supported conclusions from conjecture.

### 6.1. ISAC Sensing Preservation and Remote-Sensing Relevance

The proposed scheme is directly relevant to ISAC and remote-sensing applications. LEO satellites increasingly serve as dual-function platforms. The same downlink waveform that carries Earth-observation sensor data (optical/SAR imagery, radar products, telemetry) can simultaneously function as a sensing waveform for target parameter estimation via the AFDM delay–Doppler structure. A confidentiality mechanism that destroys the sensing function would be incompatible with such ISAC deployments.

Our time-domain mask avoids this conflict by design. Because the mask AR is a monomial (unitary) matrix, it preserves the multiset of sample magnitudes and, more importantly, the delay–Doppler matched-filter response: the key-holding receiver inverts the mask and recovers the standard AFDM effective channel $H_{\mathrm{eff}}$, whose sparse delay–Doppler support directly encodes the target scattering map. A no-key receiver instead observes the composite channel $G=H_{\mathrm{eff}}P$, where $P=DMD^{H}$ is a dense unitary matrix that smears every target peak across the full delay–Doppler grid. Section 4.6 quantifies this: sensing concentration at true target cells is 0 dB for the key holder and −16.6 dB for the no-key receiver, with 2/4 targets detectable above a −6 dB threshold for the key holder and 0/4 for the no-key receiver.

This sensing-preservation property follows from the monomial mask structure for the evaluated geometry and does not require any modification to the encryption design. It positions time-domain obfuscation as a compatible confidentiality layer for AFDM-based ISAC and remote-sensing downlinks, complementing the ISAC-focused secure AFDM work of Cao et al. [10] (which operates in the DAFT/parameter domain) with a time-domain alternative that leaves the sensing-optimal chirp parameters $c_{1},c_{2}$ unchanged.

### 6.2. Cryptographic Boundary and Nonce Requirement

The permutation R, phase mask A, and pilot phases $\left\{\theta_{p}\right\}$ are derived from a 256-bit master key via domain-separated HMAC-SM3 (hash-based message authentication code; Request for Comments (RFC) 2104) [26], placing the security boundary at the cryptographic key derivation function (KDF) rather than at a simulation-grade generator. In our current simulations R and A are static; only pilot phases would be nonce-derived via $\mathrm{HMAC}\text{-}\mathrm{SM}3(K,\;\mathrm{AFDM}\text{-}\mathrm{ENC}\text{-}\mathrm{pilot}\;|\;\underline{t}\;|\;\underline{p})$ as in (17).

The stronger-adversary experiment (Section 4.5) establishes that a single known-plaintext frame breaks composite-channel confidentiality unless pilot phases are refreshed per frame. The defense is therefore the freshness of $\left\{\theta_{p}^{\left(t\right)}\right\}$ via Equation (17). We implement and validate this defense against the cross-frame anchor attack in Section 5.12 (Figure 13): with per-frame nonces, Eve’s BER on an unknown data frame stays at ≈0.5 across all SNRs. Same-frame known-plaintext—known symbols on enough subcarriers within a single frame—remains the most important open step; same-frame decision-directed recovery is itself not identifiable by the phase-identifiability result of Section 4.5 in the constant-modulus case.

The sample mask AR is monomial and therefore linear; once Eve has formed the composite channel estimate, a known symbol on a subcarrier is observationally equivalent to a phase reference, which is the channel-coding instance of the linear-cipher weakness. Under per-frame nonces, the decision-directed and FEC-aided attacks are not identifiable by a phase-identifiability failure (analytically, under the stated constant-modulus, single-observation assumptions), but the same-frame known-plaintext boundary remains. This is a cryptographic property, not a physical-channel one. It bounds what a physical-layer mask can offer. Obfuscation against naive passive receivers (A1–A2) and cross-frame nonce defense (A4). Confidentiality against same-frame known-plaintext or coded adversaries falls to upper-layer encryption. A nonlinear sample cipher would forfeit the PAPR preservation and CPP/DAFT integration that motivate a physical-layer approach. We therefore position time-domain obfuscation as a complement to upper-layer encryption, which handles known-plaintext and coded-adversary confidentiality. The linear-mask boundary defines this division of labor.

### 6.3. Composability and Deployment

Our mask operates on time-domain samples after the IDAFT, while parameter-domain schemes act before it; the two are therefore composable, providing defense-in-depth. We do not evaluate any composition experimentally, and interactions between layers must be analyzed rather than assumed. The scheme is waveform-preserving for the legitimate receiver ($c_{1},c_{2}$, CPP, and DAFT remain standard), easing integration with an AFDM-based NTN air interface. Open integration questions include sensitivity to synchronization error and the interaction with forward error correction, which enables FEC-aided phase search—the class of attack our uncoded evaluation cannot bound.

The encryption-side operations scale linearly with the frame length and do not increase the asymptotic complexity beyond the O(NlogN) (I)DAFT that AFDM already requires: per frame the transmitter applies the O(N) sample-level mask (a permutation and *N* unit-modulus multiplications) and derives the mask material via HMAC-SM3, whose per-invocation cost is independent of *N* with the *N*-sample mask expanded from the digest. Their practical latency, energy, and hardware-resource costs remain to be measured. For the stated ordering of encryption and prefix insertion, the mask preserves the useful block’s sample magnitudes and hence its PAPR while retaining the same frame length, sampling rate, and prefix overhead; the standard AFDM structure is recovered at the legitimate receiver only after decrypt-first processing. The dominant added cost is at the legitimate receiver: the dense $O(N^{3})$ LMMSE factorization per training cycle (Table 4), whose amortized per-block cost is $O(N^{2}+N^{3}/D)$ when one factorization is reused over *D* data blocks, approaching $O(N^{2})$ only for a sufficiently long reuse interval. For a LEO downlink, the legitimate receiver, and hence this equalizer, is ground-side. If waveform generation is performed onboard, the satellite’s incremental encryption workload is limited to the O(N) mask operations; in a conventional transparent bent-pipe architecture, these operations would instead be performed at the gateway, leaving no additional satellite baseband workload. This argument does not cover uplink reception or regenerative payloads that require onboard equalization. It is a complexity- and resource-budget argument for feasibility, not a measured implementation, and it complements rather than replaces the hardware validation below.

### 6.4. Comparison with Parameter-Domain Secure-AFDM

Existing secure-AFDM schemes key the DAFT-domain chirp parameters $\left(c_{1},c_{2}\right)$. This entangles the secret with the waveform’s channel-matching design: $c_{1},c_{2}$ are confined to a narrow no-aliasing/full-diversity range, so they carry little key entropy, and keying them away from the optimal point degrades the legitimate link. Our time-domain sample mask instead leaves $\left(c_{1},c_{2}\right)$ at their channel-optimal values and draws $∼\;\log_{2}\left(N!\right)+Nb$ bits from the permutation/phase mask, decoupling security from waveform design. We also verified numerically that an equivalent-placement DAFT-domain cipher (the same mask applied before the IDAFT) matches the time-domain scheme in PAPR and composite-attack security; we adopt the time-domain placement for its exact PAPR preservation (Proposition 1) and clean decrypt-first/CPP integration. A quantitative head-to-head against CP-AFDM [14] is reported in Section 5.14.

### 6.5. Limitations and Future Work

Our security evidence consists of uncoded BER under passive adversaries A1–A4. The decision-directed and FEC-aided joint attacks are characterized analytically in Section 4.5: under per-frame nonces they are not identifiable by a phase-identifiability failure in the constant-modulus case (an analytical result under the stated assumptions, not an evaluation against an implemented decoder), and under reused pilot phases they reduce to the rotational-symmetry case. The same-frame known-plaintext boundary under nonces remains unquantified. Several other items remain open. A head-to-head comparison with CP-AFDM is reported in Section 5.14; quantitative comparisons with the other parameter-domain schemes [12,13] remain open. Reduced-pilot or compressed channel estimation would lower the training overhead. Modulation-classification and cyclostationary validation would further test the waveform obfuscation claim. The per-frame nonce should also be evaluated against same-frame known-plaintext attacks and against amplitude-varying modulation such as 16QAM, for which the constant-modulus identifiability result does not apply. The cross-frame defense is validated in Section 5.12. Other directions include extension to 3GPP NTN TDL LEO channels and deep-learning-aided equalization with adversarial detection [27]. The present study is simulation-based: it uses the NTN-motivated parameter mapping of Section 3.5 but includes no hardware or over-the-air measurement. A concrete validation roadmap would close this gap in three steps: (i) a fixed-point software-defined radio (SDR)/field-programmable gate array (FPGA) implementation of mask generation, key/frame-counter synchronization, and decrypt-first processing, measuring latency, throughput, memory, and resource usage against standard AFDM; (ii) hardware-in-the-loop testing with a channel emulator loaded with recorded or explicitly defined doubly dispersive traces, under controlled CFO, phase noise, timing error, quantization, and HPA nonlinearity; and (iii) end-to-end testing under a concrete 3GPP NTN LEO profile, e.g., a Rel-17 non-terrestrial scenario with a low-Earth-orbit satellite at approximately 600 km altitude at S- or Ka-band, an NTN-TDL channel model, and a 15–60 kHz numerology. Over-the-air testing may supplement these stages but would not by itself constitute operational LEO validation. Layered composition with DAFT-domain parameter protection (Section 6.3) is a promising direction for combining time- and parameter-domain secrecy.

Beyond the representative doubly dispersive LEO-type channel evaluated here, the evolution of non-terrestrial networks toward 6G envisions three-dimensional aerial-terrestrial integration that combines low-altitude unmanned aerial vehicles, high-altitude platform stations, and LEO satellites in a unified architecture. These environments are more dynamic and generate complex multi-layer interference. A natural extension is to make the mask adaptive or to integrate its design into a channel-model-driven optimization framework. The monomial mask preserves the PAPR of each length-*N* post-IDAFT block for any transform size *N*. Extending this property to multi-antenna or multi-link processing requires an explicit multidimensional mask construction. The present full-band estimator does not rely on channel sparsity and can represent a dense N×N effective channel under block-stationarity, but its *N*-block training requirement and dense $O(N^{3})$ equalization make rapidly varying and spatially expanded non-terrestrial channels challenging. Channel-estimate refresh, driven by the coherence time, should be distinguished from nonce-derived mask freshness, driven by the adversary model. The framework of Ma et al. [28] illustrates a broader channel-model-driven optimization methodology for layered aerial networks, but its application to Encrypted-AFDM remains to be developed and validated. We leave the full development of adaptive and channel-aware encryption for three-dimensional non-terrestrial networks to future work.

## 7. Conclusions

We investigated time-domain waveform obfuscation for AFDM, adapting two-stage unitary transformations (sample permutation and phase rotation) to the IDAFT output before CPP generation. The obfuscation masks the sample-level chirp structure while preserving PAPR exactly for the *N* post-IDAFT samples and introducing an SNR penalty below 0.5 dB for the legitimate receiver with a numerically known effective channel. A training scheme with key-derived secret pilot phases enables channel estimation and raises the cost of naive composite-channel estimation. A decrypt-first receiver architecture with numerical effective channel construction avoids the analytical channel mismatch that affected our earlier implementation.

Simulations across N=64 and N=128 with QPSK and 16QAM under integer and fractional Doppler yield four findings. A wrong-key eavesdropper on QPSK achieves uncoded BER within ∼0.01 of 0.5 across all tested SNRs. The 95% Wilson intervals sit just below 0.5 under the large trial budget, and this bias is not exploited by the naive receiver. The legitimate SNR penalty is below 0.5 dB with a numerically known effective channel. Pilot-estimated evidence is limited to QPSK at N=64. A multi-frame known-plaintext LS attack recovers the composite channel progressively, reaching BER 0.106 at *M* = *N* = 64 and 20 dB, with appreciable leakage well before that point. Blind Viterbi–Viterbi phase recovery fails under QPSK (BER 0.46–0.48), but a single known-plaintext frame breaks composite-channel confidentiality. Per-frame nonce-derived pilots are therefore mandatory. We validate this defense against the cross-frame anchor in Section 5.12.

The results support a limited claim: time-domain sample masking is a feasible confidentiality layer for AFDM. It preserves BER performance, PAPR (for the post-IDAFT samples), and receiver compatibility under the tested uncoded settings. It withstands passive receivers up to blind phase recovery when pilot phases are refreshed per frame. It does not establish secrecy against decision-directed or FEC-aided adversaries, superiority over parameter-domain schemes, or statistical noise-likeness. These are left to Section 6.5. Identifying the single-known-frame break and elevating per-frame nonces from optional hardening to a hard requirement is also a contribution, as this failure mode was not surfaced by the OFDM precedent.

Time-domain obfuscation is a new design axis for AFDM physical layer encryption, composable with existing DAFT-domain methods. Because the mask is unitary, it preserves AFDM’s delay–Doppler sensing capability for the key-holding receiver while denying it to a no-key eavesdropper, making the scheme compatible with ISAC and remote-sensing LEO downlinks. The present work establishes algorithmic feasibility under an NTN-motivated doubly dispersive channel rather than operational validation of a standards-compliant LEO link; the limitations in Section 6.5 must be addressed before operational deployment.

## Figures and Tables

**Table 1:** Positioning relative to prior secure AFDM/OFDM waveforms. A quantitative BER and PAPR comparison with CP-AFDM is reported in Section 5.14.

Scheme	Operating Domain	Security Basis	Security Evidence in Source	Main Overhead
Channel-dependent AFDM [12]	DAFT parameter $c_{2}$	Channel reciprocity	Eve BER; degrades at high Eve SNR	Reciprocity estimation
SE-AFDM [13]	DAFT parameter spreading	LPD parameter secrecy	Detection/BER analysis	Parameter signaling
CP-AFDM [14]	Chirp-subcarrier permutation	Permutation secrecy	Obfuscation analysis	Permutation key
Multi-layer AFDM-IM [10]	DAFT parameter + index modulation	Multi-dimensional key	Eve BER; ISAC sensing	Multi-parameter signaling
Secure AFDM [15]	DAFT waveform design	Waveform secrecy	BER; satellite-air link	Waveform parameters
Security-aware codebook [11]	AFDM codebook	Codebook secrecy	Eve BER; low-PAPR	Codebook distribution
Encrypted-OFDM [6]	Time-domain samples (post-IFFT)	Key-based masking	Eve BER (uncoded)	O(N) masking
This work (Enc-AFDM)	Time-domain samples (post-IDAFT)	Key-based masking + secret pilots	Uncoded Eve BER + KPA boundary	*N*-block pilot training

**Table 2:** Summary of principal notation.

Symbol	Meaning
*N*	Number of DAFT subcarriers (transform size)
x,s	DAFT-domain symbol vector, time-domain (IDAFT) samples
$c_{1},\;c_{2}$	AFDM chirp parameters (delay-shear, diversity)
$D,\;D^{-1}$	Discrete affine Fourier transform and its inverse
$F,\;\Lambda_{c_{1}},\;\Lambda_{c_{2}}$	DFT matrix, chirp diagonal matrices
$L_{\mathrm{cp}}$	Chirp-periodic prefix (CPP) length
$\alpha_{\max},\;l_{\max}$	Maximum normalized Doppler, maximum delay (samples)
*P*	Number of resolvable propagation paths
$h_{i},\;l_{i},\;\alpha_{i}$	Gain, delay, and normalized Doppler of path *i*
$H_{\mathrm{eff}}$	AFDM effective (DAFT-domain) channel matrix
${\tilde{H}}_{\mathrm{eff}}$	Post-decryption effective channel seen by Bob
G	Composite channel $D\;\circ \;H\;\circ \;{\mathrm{Enc}}_{K}\;\circ \;D^{-1}$ seen by Eve
K,t	Shared secret key, per-frame nonce
R,A	Sample-permutation and diagonal phase-mask matrices
${\mathrm{Enc}}_{K},\;{\mathrm{Dec}}_{K}$	Time-domain encryption/decryption maps AR, $R^{-1}A^{H}$
$\left\{\theta_{p}^{\left(t\right)}\right\}$	Key-derived secret pilot phases for frame *t*
K	Mask configuration space, $\left|K\right|=N!\;2^{bN}$
$M,\;N_{d}$	Modulation order, number of active data subcarriers
$\lambda ,\;\sigma^{2}$	LMMSE regularization, noise variance
A	Adversary class (A1–A4)

**Table 3:** Illustrative dimensional mapping of NTN-motivated link quantities [9] to the normalized simulation parameters, for the default N=64 integer-Doppler communication experiments (fractional-Doppler and dedicated sensing experiments use larger Doppler extents, as noted in Section 5). This is a dimensional interpretation, not a standards-compliant NTN link.

NTN-Motivated Quantity	Illustrative Value	Simulation Parameter
Altitude/velocity	300–1500 km/7.5 km/s	scenario geometry
Carrier band	Ka-band (20–30 GHz)	Doppler budget
Residual Doppler (stress-test bound)	up to 1 SCS	$\alpha_{\max}=1$
Subcarrier spacing (NR numerology)	240 kHz (illustrative)	Δf
Nominal *N*-tone bandwidth (*N* = 64)	≈15.4 MHz	$T_{s}\approx 65$ ns
Maximum modeled excess delay	≈390 ns	$l_{\max}=6$ samples
Channel profile	6-tap exponential AFDM benchmark	not a 3GPP NTN-TDL

**Table 4:** Per-block computational complexity (complex multiplications).

Stage	OFDM	AFDM	Enc-AFDM
(I)DAFT/(I)DFT	O(NlogN)	O(NlogN)+2N	O(NlogN)+2N
Time mask AR	–	–	*N* (+O(N) perm.)
Key derivation	–	–	O(N)
Equalization	O(N)	O(N)	$O(N^{3})$/$O(N^{2})\;$ ^†^
Eve (A2–A4)	–	–	$O(N^{3}+KN)$

^†^ $O(N^{3})$ factorization per training cycle; $O(N^{2}+N^{3}/D)$ per data block thereafter (factorization amortized over *D* blocks).

**Table 5:** Adversary classes evaluated in this work.

Class	Capability	Exp.
A1	Wrong-key decrypt-then-LMMSE chain	Section 4.2 and Section 5.3
A2	Composite-channel estimate from secret pilots, no decryption	Section 4.3, Section 5.10 and Section 5.11
A3	Multi-frame known-plaintext LS recovery from data frames	Section 4.4 and Section 5.9
A4	Composite estimate GΘ + blind Viterbi–Viterbi phase recovery (*K* frames); optional single known-plaintext anchor	Section 4.5, Section 5.11 and Section 5.12

**Table 6:** Simulation parameters for Encrypted-AFDM experiments.

Parameter	Value
FFT size *N*	64 (baseline), 128 (scalability test)
Maximum Doppler $\alpha_{\max}$	1
Maximum delay $l_{\max}$	6 samples
CPP length $L_{\mathrm{cp}}$	6
Chirp parameters	$c_{1}=3/\left(2N\right)$, $c_{2}=\sqrt{2}/N$
Guard symbols per side	$\alpha_{\max}$ (integer), $\alpha_{\max}+\left\lceil \xi \right\rceil$ (fractional)
Modulation	QPSK, 16QAM
SNR range	0–20 dB (0.5 dB step)
Trials per SNR	200–2000
Channel model	6-tap Rayleigh, exp. power delay profile
Path losses (dB)	[0,−3.6,−7.2,−10.8,−18.0,−25.2]
Delays (samples)	[0,1,2,3,5,6]
Doppler	$U\{-\alpha_{\max},\alpha_{\max}\}$ (int), +U(−ξ,ξ) (frac)
Fractional Doppler ξ	0.3 (scalability test)
Master key (Bob)	256-bit HMAC-SM3
Master key (Eve)	256-bit HMAC-SM3 (independent)
Phase resolution	8-bit per sample (256 levels per sample)
LMMSE regularization λ	1

**Table 7:** Representative two-sided 95% Wilson score intervals for security-critical BER configurations (N=64, QPSK, 20 dB). Zero-error points use the one-sided 95% upper bound. Pooled bit counts are approximate.

Configuration	$\hat{p}$	Pooled Bits *n*	95% Wilson Interval
E1 wrong-key Eve	0.488	∼$2.5\;\times \;10^{5}$	[0.4860,0.4900]
E7 KPA, *M* = *N* = 64	0.106	∼$1.2\;\times \;10^{3}$	[0.090,0.124]
E8 key-reuse	0.463	∼$5.0\;\times \;10^{3}$	[0.449,0.477]
E9 single known frame	$4.2\;\times \;10^{-3}$	$4.96\;\times \;10^{3}$	[0.0027,0.0064]
E10 nonce defense	0.52	∼$4.0\;\times \;10^{3}$	[0.505,0.535]
Bob genie, zero-error high-SNR point	0	∼$2.5\;\times \;10^{5}$	one-sided upper ≈$1.1\;\times \;10^{-5}$

**Table 8:** Low-entropy ablation (BER at 20 dB SNR).

Configuration	Bob BER	Eve BER
None	$1.0\times 10^{-4}$	$1.0\times 10^{-4}$
Scramble-only	$1.0\times 10^{-4}$	0.496
Phase-only	$1.0\times 10^{-4}$	0.500
Both	$1.0\times 10^{-4}$	0.502

## Data Availability

The MATLAB simulation code and the generated data that support the findings of this study will be made openly available in a public repository upon publication, and are available from the corresponding author upon reasonable request in the interim.

## Abbreviations

The following abbreviations are used in this manuscript:

AFDM	Affine frequency division multiplexing
CCDF	Complementary cumulative distribution function
CoV	Coefficient of variation
CP-AFDM	Chirp-permuted AFDM
CPP	Chirp-periodic prefix
DAFT	Discrete affine Fourier transform
EVM	Error vector magnitude
HMAC	Hash-based message authentication code
HPA	High-power amplifier
IDAFT	Inverse discrete affine Fourier transform
ISAC	Integrated sensing and communication
JSR	Jammer-to-signal ratio
KDF	Key derivation function
KPA	Known-plaintext attack
LEO	Low Earth orbit
LMMSE	Linear minimum mean-square error
LPD	Low probability of detection
NMSE	Normalized mean-square error
NR	New Radio (5G)
NTN	Non-terrestrial network
PAPR	Peak-to-average power ratio
PLE	Physical layer encryption
PLS	Physical layer security (information-theoretic)
PRF	Pseudo-random function
SCS	Subcarrier spacing
TDL	Tapped delay line
V&V	Viterbi–Viterbi (fourth-power phase estimator)
